# Supplementary material for: Effect of COVID-19 lockdown on hospital admissions and mortality in rural KwaZulu-Natal, South Africa: interrupted time series analysis
Source: BMJ Open. 2021 Mar 18;11(3):e047961. doi: 10.1136/bmjopen-2020-047961 (PMC7977076; doi:10.1136/bmjopen-2020-047961)

Supplementary Figure 1. LOWESS curves of daily admissions by age and sex, 2019-2020

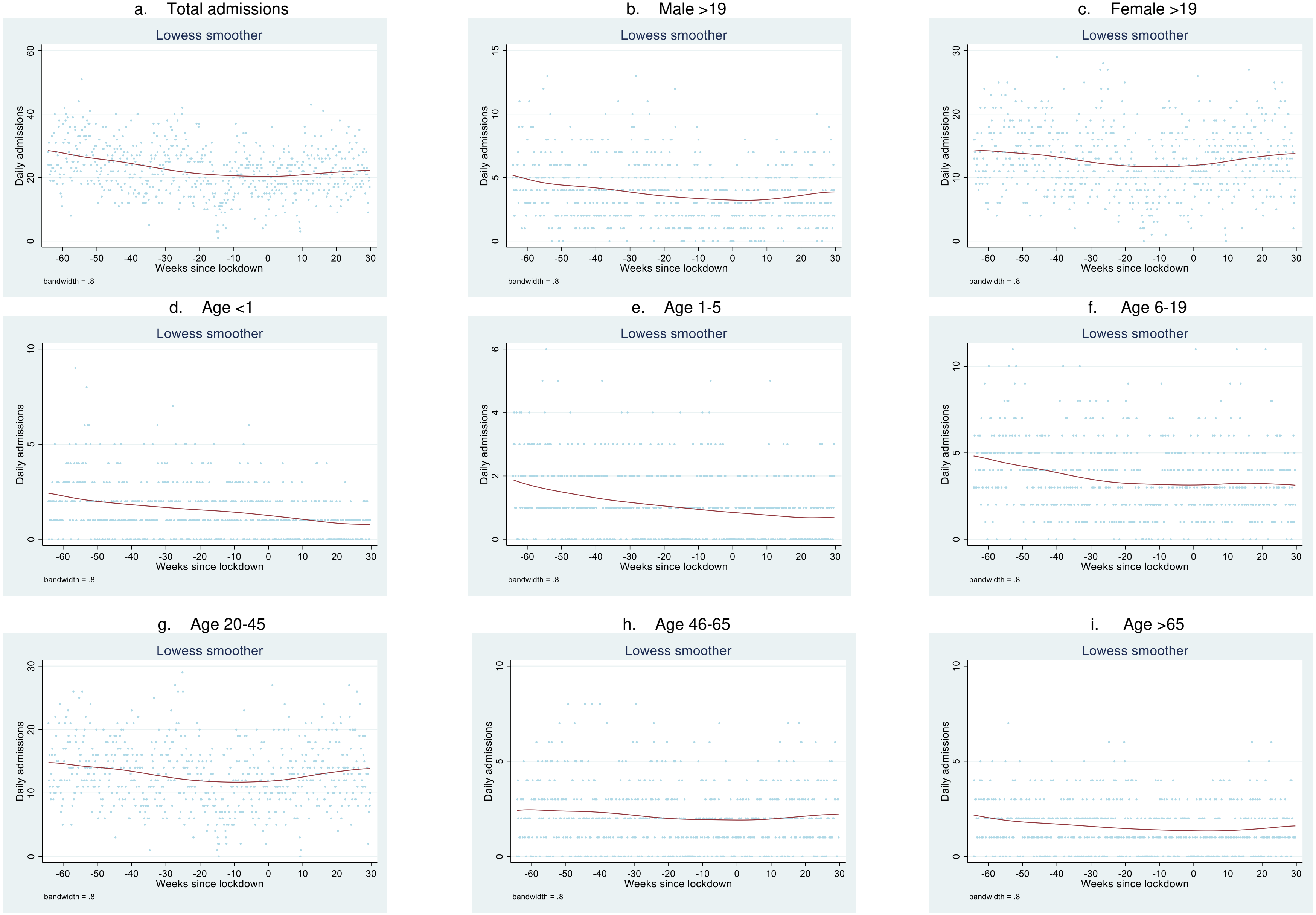

BMJ Publishing Group Limited (BMJ) disclaims all liability and responsibility arising from any reliance placed on this supplemental material which has been supplied by the author(s)

Supplementary Figure 2. LOWESS curves of daily admissions by diagnostic category, 2019-2020

a. Maternal and neonatal

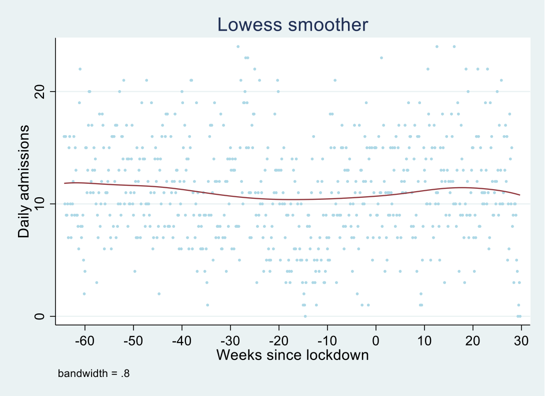

b. Non-communicable diseases

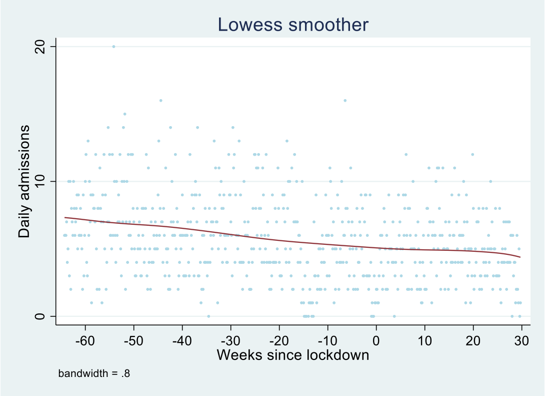

c. Communicable diseases

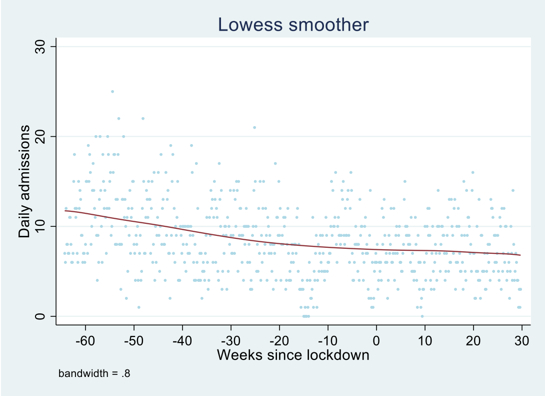

d. Injuries

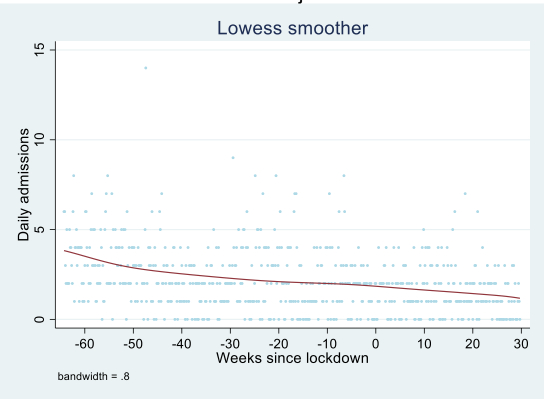

e. Respiratory

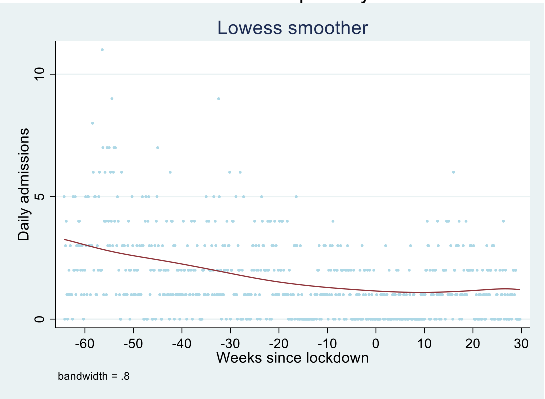

Supplementary Figure 3. LOWESS curve of probability of death by week of admission

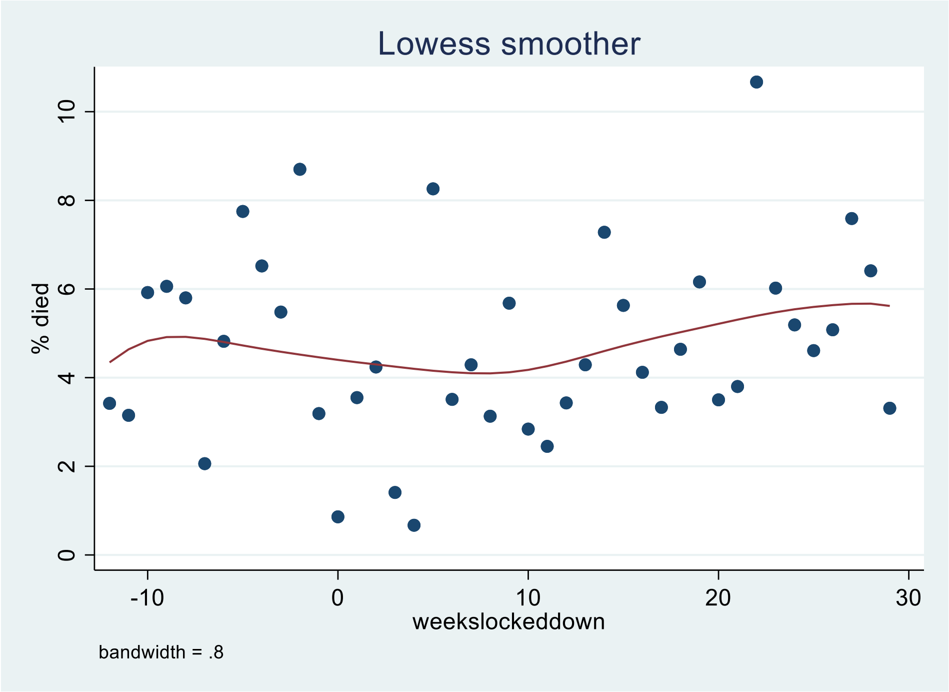

Supplement: Supplementary data [file bmjopen-2020-047961supp001.pdf]
