# Supplementary material for: Effect of COVID-19 lockdown on hospital admissions and mortality in rural KwaZulu-Natal, South Africa: interrupted time series analysis
Source: BMJ Open. 2021 Mar 18;11(3):e047961. doi: 10.1136/bmjopen-2020-047961 (PMC7977076; doi:10.1136/bmjopen-2020-047961)
Supplement: Supplementary data [file bmjopen-2020-047961supp002.pdf]

Supplementary Table 1: Changes in mean daily admissions to Hlabisa hospital at each stage of lockdown: linear regression models adjusted for day of week

	Pre-lockdown average daily admissions (95% CI)			Change at level 5	95% CI		P value	Change at level 4	95% CI		P value	Change at level 3	95% CI		P value	Change at level 2	95% CI		P value	Change at level 1	95% CI		P value
All admissions	16.96	14.93	19.00	-0.74	-3.02	1.53	0.52	-0.23	-3.03	2.57	0.87	3.18	0.77	5.59	0.01	0.40	-1.94	2.73	0.74	-1.88	-4.72	0.97	0.20
Male >19	2.55	1.85	3.26	-0.13	-0.92	0.66	0.74	-0.20	-1.17	0.77	0.68	1.27	0.44	2.11	<0.001	-0.07	-0.88	0.74	0.87	-0.25	-1.24	0.73	0.61
Female >19	8.70	7.17	10.23	0.54	-1.17	2.26	0.53	0.33	-1.78	2.44	0.76	1.36	-0.46	3.17	0.14	0.51	-1.25	2.27	0.57	-1.17	-3.31	0.97	0.28
Age <1	1.55	1.19	1.91	-0.64	-1.04	-0.23	<0.001	-0.44	-0.93	0.06	0.08	0.34	-0.09	0.76	0.12	-0.25	-0.66	0.17	0.24	0.12	-0.38	0.62	0.64
Age 1-5	1.05	0.71	1.39	-0.50	-0.88	-0.12	0.01	-0.07	-0.53	0.40	0.78	0.27	-0.13	0.67	0.19	-0.36	-0.74	0.03	0.07	0.35	-0.13	0.82	0.15
Age 6-19	3.11	2.41	3.81	-0.02	-0.80	0.76	0.96	0.14	-0.82	1.11	0.77	-0.05	-0.88	0.77	0.90	0.56	-0.25	1.36	0.17	-0.92	-1.90	0.06	0.07
Age 20-45	8.89	7.37	10.40	0.87	-0.82	2.56	0.31	-0.55	-2.63	1.53	0.60	2.14	0.34	3.93	0.02	0.72	-1.01	2.46	0.41	-1.44	-3.55	0.68	0.18
Age 46-65	1.23	0.71	1.75	-0.20	-0.78	0.38	0.50	0.37	-0.34	1.09	0.31	0.43	-0.18	1.05	0.17	-0.54	-1.13	0.06	0.08	0.30	-0.43	1.03	0.42
Age >65	1.14	0.70	1.57	-0.26	-0.74	0.23	0.30	0.31	-0.29	0.90	0.31	0.06	-0.46	0.57	0.83	0.26	-0.24	0.75	0.31	-0.29	-0.90	0.31	0.35
Maternal and neonatal	8.10	6.61	9.60	0.90	-0.77	2.57	0.29	0.47	-1.58	2.53	0.65	0.31	-1.46	2.08	0.73	0.99	-0.72	2.70	0.26	-3.33	-5.41	-1.24	0.002
NCDs	3.84	2.96	4.72	-0.48	-1.47	0.50	0.34	0.65	-0.56	1.87	0.29	0.37	-0.67	1.41	0.49	-0.53	-1.54	0.48	0.30	-1.23	-2.46	-0.001	0.05
CDs	6.49	5.38	7.60	-0.94	-2.18	0.30	0.14	-0.32	-1.84	1.21	0.68	0.97	-0.34	2.29	0.15	-0.73	-2.00	0.55	0.26	-1.04	-2.59	0.51	0.19
Injuries	2.47	1.98	2.96	-0.41	-0.96	0.14	0.15	-0.27	-0.94	0.41	0.44	0.46	-0.13	1.04	0.13	-0.22	-0.78	0.35	0.45	-0.74	-1.43	-0.06	0.03
Respiratory	0.73	0.35	1.10	-0.49	-0.91	-0.08	0.02	-0.26	-0.77	0.25	0.32	1.27	0.83	1.71	<0.001	-0.70	-1.12	-0.27	0.002	-0.11	-0.63	0.41	0.68

NCDs: non-communicable disease

CDs: communicable diseases

CI: confidence interval

Supplementary Table 2: Time trends and step changes in daily hospital admissions before and after start of level 5 lockdown: Poisson regression models adjusted for day of week

	Pre-lockdown daily incidence rate	95% Confidence Interval		IRR pre-lockdown (per week)	95% Confidence Interval		P value	IRR at lockdown	Confidence Interval		P value	IRR post-lockdown (per week)	95% Confidence Interval		P value	P value of interaction term
All admissions	16.06	14.30	18.03	0.990	0.977	1.003	0.14	1.06	0.95	1.19	0.31	1.004	1.000	1.007	0.04	0.05
Male >19	1.71	1.24	2.36	0.94	0.91	0.97	<0.001	1.50	1.10	2.06	0.01	1.011	1.002	1.020	0.01	<0.001
Female>19	9.23	7.92	10.76	1.01	0.99	1.02	0.57	1.05	0.91	1.21	0.51	1.004	1.000	1.009	0.06	0.92
Age <1	1.12	0.71	1.75	0.96	0.92	1.004	0.08	0.74	0.48	1.16	0.19	0.99	0.98	1.01	0.45	0.16
Age 1-5	0.69	0.40	1.19	0.94	0.89	0.998	0.04	0.88	0.51	1.52	0.65	1.00	0.98	1.02	0.85	0.05
Age 6-19	3.50	2.65	4.62	1.02	0.99	1.05	0.26	0.96	0.73	1.26	0.76	1.00	0.99	1.01	0.53	0.21
Age 20-45	9.17	7.86	10.69	1.00	0.98	1.02	0.91	1.08	0.93	1.25	0.29	1.005	1.0003	1.01	0.04	0.69
Age 46-65	1.01	0.67	1.52	0.96	0.92	1.01	0.10	1.34	0.91	1.96	0.14	1.006	0.995	1.018	0.26	0.06
Age >65	0.82	0.51	1.32	0.95	0.90	1.0003	0.05	1.23	0.77	1.96	0.39	1.012	0.998	1.026	0.08	0.02
Maternal and neonatal	9.67	8.27	11.30	1.03	1.01	1.05	0.01	1.01	0.87	1.18	0.86	0.997	0.992	1.002	0.21	0.003
NCDs	3.27	2.57	4.18	0.97	0.95	1.001	0.06	1.26	0.998	1.58	0.05	0.995	0.988	1.002	0.19	0.14
CDs	5.52	4.55	6.00	0.98	0.96	0.998	0.04	1.07	0.89	1.28	0.47	0.997	0.991	1.003	0.29	0.08
Injuries	1.83	1.26	2.00	0.95	0.91	0.99	0.02	1.29	0.88	1.89	0.19	0.986	0.974	0.999	0.04	0.09
Respiratory	0.40	0.23	0.71	0.92	0.87	0.97	0.004	1.25	0.73	2.12	0.41	1.02	1.002	1.03	0.03	0.001

NCDs: non-communicable disease

CDs: communicable diseases

 BMJ Publishing Group Limited (BMJ) disclaims all liability and responsibility arising from any reliance placed on this supplemental material which has been supplied by the author(s)

Supplementary Table 3. Time trends and step changes in daily hospital admissions before and after start of level 5 lockdown: linear regression models adjusted for day of week

	Average admissions day before lockdown	95% Confidence Interval		Change in daily admissions/week pre-lockdown	95% Confidence Interval		P value	Change in daily admissions at lockdown	95% Confidence Interval		P value	Change in daily admissions/week post-lockdown	95% Confidence Interval		P value	P value of interaction term
All admissions	15.71	12.75	18.66	-0.20	-0.55	0.15	0.26	1.17	-1.76	4.10	0.43	0.08	-0.02	0.17	0.10	0.13
Male >19	1.42	0.42	2.43	-0.18	-0.30	-0.06	0.003	1.08	0.08	2.08	0.03	0.04	0.00	0.07	0.02	0.001
Female>19	9.03	6.82	11.24	0.06	-0.20	0.32	0.67	0.58	-1.61	2.77	0.60	0.05	-0.02	0.12	0.13	0.99
Age <1	1.11	0.60	1.63	-0.07	-0.13	-0.01	0.02	-0.31	-0.82	0.20	0.23	-0.01	-0.02	0.01	0.48	0.04
Age 1-5	0.64	0.16	1.13	-0.07	-0.12	-0.01	0.02	-0.05	-0.53	0.43	0.85	0.001	-0.01	0.02	0.87	0.03
Age 6-19	3.49	2.49	4.50	0.06	-0.06	0.18	0.31	-0.14	-1.13	0.86	0.79	-0.01	-0.04	0.02	0.57	0.26
Age 20-45	8.92	6.73	11.11	0.01	-0.25	0.27	0.95	0.92	-1.26	3.09	0.41	0.06	-0.01	0.13	0.08	0.70
Age 46-65	0.82	0.06	1.57	-0.07	-0.16	0.02	0.13	0.50	-0.24	1.24	0.19	0.01	-0.01	0.04	0.28	0.08
Age >65	0.72	0.10	1.34	-0.07	-0.14	0.00	0.07	0.24	-0.37	0.85	0.44	0.02	0.00	0.04	0.09	0.03
Maternal and neonatal	9.74	7.58	11.89	0.27	0.01	0.52	0.04	0.23	-1.91	2.36	0.83	-0.03	-0.10	0.03	0.32	0.03
Non-communicable diseases	3.07	1.79	4.34	-0.13	-0.28	0.02	0.09	1.11	-0.15	2.38	0.08	-0.02	-0.06	0.02	0.24	0.19
Communicable diseases	5.38	3.78	6.97	-0.18	-0.37	0.01	0.06	0.54	-1.04	2.12	0.50	-0.02	-0.07	0.03	0.37	0.11
Injuries	1.83	1.13	2.54	-0.11	-0.19	-0.02	0.01	0.46	-0.24	1.16	0.20	-0.02	-0.04	0.001	0.06	0.06
Respiratory	0.14	-0.42	0.71	-0.09	-0.16	-0.03	0.01	0.23	-0.32	0.79	0.41	0.02	0.000	0.036	0.04	0.001

Supplementary Table 4: Risk of death among admissions: logistic regression models with and without adjustment for sex, age category and diagnosis.*

	Odds ratio	95% Confidence Interval		P value
**Model 1, not adjusted for case mix**				
Weekly change in risk of death pre-lockdown	1.03	0.97	1.09	0.37
Step change in risk of death at lockdown	0.49	0.29	0.82	0.01
Weekly change in risk of death post-lockdown	1.03	1.01	1.04	0.002*
**Model 2, adjusted for case mix**				
Weekly change in risk of death pre-lockdown	1.10	1.02	1.18	0.01
Step change in risk of death at lockdown	0.31	0.17	0.56	<0.001
Weekly change in risk of death post-lockdown	1.03	1.01	1.05	0.001*
Female	0.73	0.56	0.94	0.02
Age	1.02	1.02	1.03	<0.001
Communicable disease	2.33	1.46	3.71	<0.001
Neoplasm	3.32	1.52	7.26	0.003
Blood disorder	1.96	1.01	3.83	0.05
Endocrine	0.95	0.48	1.90	0.89
Mental health	0.41	0.13	1.35	0.14
Neurological	2.33	0.99	5.49	0.05
Otological	2.59	0.33	20.63	0.37
Circulatory	3.11	1.84	5.24	<0.001
Respiratory	2.47	1.49	4.08	<0.001
Gastroenterology	2.75	1.42	5.31	0.003
Dermatology	0.73	0.30	1.80	0.49
Musculoskeletal	0.49	0.07	3.62	0.49
Renal and urology	1.48	0.78	2.82	0.23
Breast and gynaecology	0.27	0.04	2.06	0.21
Maternal	1.00	0.00	0.00	<0.001
Neonatal	2.31	1.15	4.66	0.02
Injuries	0.31	0.09	1.04	0.06

* Lockdown-time interaction: P=0.325 for model 1 and P=0.126 for model 2
