# Supplementary material for: Effect of COVID-19 lockdown on hospital admissions and mortality in rural KwaZulu-Natal, South Africa: interrupted time series analysis
Source: BMJ Open. 2021 Mar 18;11(3):e047961. doi: 10.1136/bmjopen-2020-047961 (PMC7977076; doi:10.1136/bmjopen-2020-047961)
Supplement: Author's manuscript [file bmjopen-2020-047961.draft_revisions.pdf]

BMJ Open is committed to open peer review. As part of this commitment we make the peer review history of every article we publish publicly available.

When an article is published we post the peer reviewers' comments and the authors' responses online. We also post the versions of the paper that were used during peer review. These are the versions that the peer review comments apply to.

The versions of the paper that follow are the versions that were submitted during the peer review process. They are not the versions of record or the final published versions. They should not be cited or distributed as the published version of this manuscript.

BMJ Open is an open access journal and the full, final, typeset and author-corrected version of record of the manuscript is available on our site with no access controls, subscription charges or pay-per-view fees (http://bmjopen.bmj.com).

If you have any questions on BMJ Open's open peer review process please email info.bmjopen@bmj.com

BMJ Open

Effect of COVID-19 lockdown on hospital admissions and mortality in rural KwaZulu-Natal, South Africa: interrupted time series analysis

Journal:	*BMJ Open*
Manuscript ID	bmjopen-2020-047961
Article Type:	Original research
Date Submitted by the Author:	13-Dec-2020
Complete List of Authors:	McIntosh, Amy; University of East Anglia Faculty of Medicine and Health Sciences,<br>Bachmann, Max; University of East Anglia Faculty of Medicine and Health Sciences<br>Siedner, Mark; Africa Health Research Institute; Harvard Medical School<br>Gareta, Dickman; Africa Health Research Institute<br>Seeley, Janet; Africa Health Research Institute; London School of Hygiene and Tropical Medicine, Global Health and Development<br>Herbst, Kobus; Africa Health Research Institute
Keywords:	PUBLIC HEALTH, COVID-19, PAEDIATRICS

SCHOLARONE™
Manuscripts

I, the Submitting Author has the right to grant and does grant on behalf of all authors of the Work (as defined in the below author licence), an exclusive licence and/or a non-exclusive licence for contributions from authors who are: i) UK Crown employees; ii) where BMJ has agreed a CC-BY licence shall apply, and/or iii) in accordance with the terms applicable for US Federal Government officers or employees acting as part of their official duties; on a worldwide, perpetual, irrevocable, royalty-free basis to BMJ Publishing Group Ltd ("BMJ") its licensees and where the relevant Journal is co-owned by BMJ to the co-owners of the Journal, to publish the Work in this journal and any other BMJ products and to exploit all rights, as set out in our licence.

The Submitting Author accepts and understands that any supply made under these terms is made by BMJ to the Submitting Author unless you are acting as an employee on behalf of your employer or a postgraduate student of an affiliated institution which is paying any applicable article publishing charge ("APC") for Open Access articles. Where the Submitting Author wishes to make the Work available on an Open Access basis (and intends to pay the relevant APC), the terms of reuse of such Open Access shall be governed by a Creative Commons licence – details of these licences and which Creative Commons licence will apply to this Work are set out in our licence referred to above.

Other than as permitted in any relevant BMJ Author's Self Archiving Policies, I confirm this Work has not been accepted for publication elsewhere, is not being considered for publication elsewhere and does not duplicate material already published. I confirm all authors consent to publication of this Work and authorise the granting of this licence.

For peer review only

Effect of COVID-19 lockdown on hospital admissions and mortality in rural KwaZulu-Natal, South Africa: interrupted time series analysis

AUTHORS

Amy McIntosh

Norwich Medical School, Faculty of Medicine and Health Science, University of East Anglia, Norwich, UK

https://orcid.org/0000-0003-3690-2914

Max Bachmann

Norwich Medical School, Faculty of Medicine and Health Science, University of East Anglia, Norwich, UK

https://orcid.org/0000-0003-1770-3506

Mark J Siedner

Africa Health Research Institute, KwaZulu-Natal, South Africa

Department of Medicine, Harvard Medical School, Boston, Massachusetts, USA

http://orcid.org/0000-0003-3506-842X

Dickman Gareta

https://orcid.org/0000-0002-4004-5655

Africa Health Research Institute, Kwa-Zulu Natal, South Africa

Janet Seeley

Africa Health Research Institute, KwaZulu-Natal, South Africa

Department of Global Health and Development, London School of Hygiene and Tropical Medicine, London, UK

https://orcid.org/0000-0002-0583-5272

Kobus Herbst

Africa Health Research Institute, KwaZulu-Natal, South Africa

DSI-MRC South African Population Research Infrastructure Network, South Africa.

https://orcid.org/0000-0002-5436-9386

Corresponding Author: Dr Amy McIntosh, A.Mcintosh@doctors.org.uk

Word Count:3392

For peer review only - http://bmjopen.bmj.com/site/about/guidelines.xhtml

ABSTRACT

Objective Assess the effect of lockdown during the 2020 COVID-19 pandemic on daily admissions and odds of death in a hospital in rural KwaZulu-Natal (KZN).

Design Observational cohort.

Setting Referral hospital for 17 primary care clinics in uMkhanyakude district.

Participants 5848 patients contributed to 6173 admissions from 1st January to 20th October 2020.

Exposure Five levels of national lockdown in South Africa from 27th March 2020, with restrictions decreasing from levels 5 to 1 respectively.

Outcome Measures Changes and trends in daily admissions and risk of in-hospital mortality before, and at each stage of lockdown, estimated by interrupted time series regression, with stratification for age, sex, and diagnosis; odds of death in hospital.

Results Daily admissions decreased during level 5 lockdown for infants (incidence rate ratio (IRR) compared to pre-lockdown 0.63, 95% confidence interval (CI) 0.44-0.90), 1-5 year-olds (IRR 0.43, 95% CI 028-0.65) and for respiratory diagnoses (IRR 0.57,95% CI 0.36-0.90). From level 4 to level 3, total admissions increased (IRR 1.17, 95% CI 1.06-1.28) as well as for men >19 years (IRR 1.50, 95% CI 1.17-1.92) and respiratory diagnoses (IRR 4.26, 95% CI 2.36-7.70). Among patients admitted to hospital, odds of death decreased during level 5 compared to pre-lockdown (adjusted odds ratio (aOR) 0.48; 95% CI 0.28-0.83), then increased from level 5 to level 4 (aOR 1.94, 95% CI 1.01-3.71) and increased again from level 3 to level 2 (OR 1.74, 95% CI 1.15-2.63).

Conclusions Level 5 lockdown is likely to have prevented the most vulnerable population, children <5 years and those more severely ill, accessing hospital care in rural KZN, as reflected by the drop in admissions and odds of mortality. Subsequent increases in admissions, and in odds of death in hospital, could be due to improved and delayed access to hospital as restrictions were eased.

KEYWORDS

COVID-19, universal healthcare, secondary care, hospital admissions, healthcare utilisation, public health

For peer review only - http://bmjopen.bmj.com/site/about/guidelines.xhtml

STRENGTHS AND LIMITATIONS OF THIS STUDY

- This paper provides original evidence of impact of COVID-19 lockdown on hospital admissions and mortality in South Africa, using diagnostic and mortality data prospectively collected and coded by trained nurse researchers
- Hospital data provide only indirect evidence of changing needs for and access to hospital care in the surrounding population
- Restriction of data to one hospital limits generalisability, and statistical power for subgroup analyses

For peer review only

BACKGROUND

The COVID-19 global pandemic has caused huge disruption to health systems worldwide. As numbers of cases of the novel coronavirus rose throughout 2020, health systems responded by focussing resources onto managing COVID-19. This led to global changes in resource allocation for non-COVID-19 services [1–4]. Concurrently, governments attempted to reduce community transmission with lockdowns, bringing in restrictions on movement and activities. Such restrictions have had far-reaching impacts on all aspects of society, including economies, education, unemployment, inequalities and health[5–7].

The effects of these measures on progress to reach universal health coverage globally are largely unknown, but it is likely that progress in low and middle income countries was adversely affected due to weak and under-resourced health systems serving populations with high burdens of both communicable (CDs) and non-communicable disease (NCDs), as well as social and economic deprivation [8]. Concerns have been voiced about impacts on HIV care, family planning services, and maternal and child health [9–11].

An unintended potential consequence of lockdowns is reduced utilisation amongst people needing health care for non-COVID-19 conditions. Excess mortality during the pandemic that is not explained by direct mortality from COVID-19 supports this concern [12,13]. Predicted effects, both through reduced health service provision and reduced utilisation, include higher rates of mortality from vaccine-preventable disease, HIV, tuberculosis, in children aged under five years and pregnant women [14–17].

South Africa implemented a level 5 lockdown order at midnight 26th March 2020 to prevent community transmission of COVID-19 [18]. This prohibited non-essential movement outside of the home across the country, and closure of non-essential businesses, schools and services. Health workers were exempt from movement restrictions, as was accessing health services. However, this led to significant reductions in availability of transport, and reductions to income, particularly for informal or non-essential workers. Restrictions were eased to level 4 lockdown from 1st May – 31st May 2020, which included some workplaces reopening[19]. This was further eased to level 3 from 1st June – 17th August, with return to work, and public transport with physical distancing and mask-wearing[20]. Level 2 was from 18th August – 20th October[21], with gatherings of less than 50 people, a wider range of leisure and social activities allowed, and alcohol sale permitted. The lowest level of lockdown, level 1, was initiated on 21st September[22] and continued beyond the end of this study (20th October 2020).

The same lockdown restrictions applied to the whole country, including KwaZulu-Natal province, where reported COVID-19 cases and deaths only began to increase after the end

For peer review only - http://bmjopen.bmj.com/site/about/guidelines.xhtml

of May[23]. Reported COVID-19 cases in KwaZulu-Natal peaked around the 25th July, and COVID-19 deaths peaked around 8th August. By 20th October reported daily COVID-19 cases and deaths in the province had decreased to less than 10 and 1 per million population, respectively. The effects of levels 5, 4 and 3 lockdown on utilisation of primary care facilities in the mostly rural KwaZulu-Natal subdistrict where the present study was conducted have been reported by Siedner and colleagues[24]. They found that level 5 lockdown was associated with a significant reduction in visits to primary care clinics for child health and in under 5 year-olds, but not for total visits or for adult care.

In the present study we investigated the effects of the lockdown measures during the COVID-19 pandemic on admissions to, and deaths in, the referral hospital serving the same clinics and population as the latter study. We focussed particularly on admissions in sub-groups stratified by age, sex and diagnosis, as indicators of access to hospital care. We also compared the risk of in-hospital mortality before and after lockdown as an indicator of the severity of presenting illnesses. We hypothesised that lockdown measures would lead to reduced hospital admissions, because of reduced mobility, and would increase as lockdown restrictions were eased. We also hypothesised that risk of in-hospital mortality would increase if people with less severe illness were selectively deterred from going to hospital, or would decrease if those with more severe illness were selectively deterred.

METHODS

Study Population

We performed secondary analysis of data collected by the Africa Health Research Institute (AHRI), located in a sub-district of the northern KwaZulu-Natal, uMkhanyakude district in South Africa. It is a largely rural district, which is socio-economically deprived, falling in the bottom 10 of all districts in South Africa for education levels, employment, access to piped water and sanitation in the home[25]. The study population was patients admitted to Hlabisa hospital from 1st January 2020 to 20th October 2020. The hospital is the main referral hospital for 17 clinics in the sub-district and is in an urban centre. Only 5% of the population hold medical insurance and so are mostly dependent on the hospital and surrounding clinics for their health care[25].

Data Collection

Data for each admission were captured prospectively by AHRI clinical research workers based in the hospital, including diagnostic coding by a trained professional nurse. These include demographic data for each patient, as well as admission, discharge, and death diagnoses, coded according to International Classification of Disease (ICD-10) classification,

For peer review only

and recorded on an electronic database[26]. Women admitted for normal vaginal deliveries were not included, but women admitted for elective Caesarean-section, antenatal care, or other intrapartum care were included.

Statistical Analysis

Frequencies and proportions of admitted patients, stratified by age, sex and discharge diagnosis were calculated. We calculated the number of admissions each day. To identify suitable regression models for estimating effects of lockdown we first graphically plotted numbers of daily admissions and probabilities of death over time with LOWESS smoothing.

Interrupted time series linear regression was used to estimate the effects of lockdown on numbers of daily admissions to hospital. We first calculated the number of admissions each day then analysed the aggregated data at day level. These analyses were carried out for all admissions from 1st January 2020 to 20th October 2020, and then repeated in subgroups defined by age, sex and diagnosis. We used two sets of models, one estimating step changes in daily admissions or odds of death during each level of lockdown compared to pre-lockdown, and the other estimating linear time trends over the entire lockdown period.

To estimate absolute changes in mean numbers of daily admissions between each level of lockdown, compared to the pre-lockdown period, we used the following model:

$$Yt = \beta_0 + \beta_1 L_5 + \beta_2 L_4 + \beta_3 L_3 + \beta_4 L_2 + \beta_5 L_1 + \beta_{6-11} Z$$

Yt is the daily number of hospital admissions. β_0 is the mean daily number of visits or admissions immediately before lockdown (the regression intercept). L_5, L_4, L_3, L_2 *and* L_1 are binary dummy variables indicating each stage of lockdown, and their coefficients ($\beta_1 - \beta_5$) are mean differences from the pre-lockdown period; differences between successive levels were the differences between the corresponding coefficients. Z is a vector of dummy variables representing day of week.

The regression equation to estimate the continuous time trend before and after level 5 lockdown, and step change at the start of level 5, was as follows:

$$Yt = \beta_0 + \beta_1 T + \beta_2 X_t + \beta_3 T X_t + \beta_4 Z$$

Yt, β_0, and Z are the same as in the previous equation. β_1 is the weekly change in daily admissions before lockdown (that is, coefficient of the slope before lockdown) and T represents the number of weeks before or after lockdown. β_2 is the step change in number of admissions immediately after level 5 lockdown on 27th March 2020. X_t represents the pre- and post-lockdown periods coded as a binary variable. β_3 is the change in trend after

For peer review only - http://bmjopen.bmj.com/site/about/guidelines.xhtml

lockdown. TX_t is the time-lockdown interaction term; $\beta_1 + \beta_3$ is thus the time trend after lockdown. We used the estimated values from each analysis to graphically plot predicted continuous time trends and step changes.

We first used Poisson regression to estimate relative changes and then used linear regression to estimate absolute changes in daily admissions associated with lockdown. We plotted frequency distributions of residuals of linear regression models, overall and in subgroups, to assess their normality. Results of the linear regression models with continuous time trends were used to plot graphs of predicted values associated with numbers of weeks before and after lockdown.

Odds of in-hospital mortality among admitted patients was investigated using equivalent logistic regression models (with Yt representing log odds of death). These analyses were at the level of individual admission, with robust adjustment for intra-person correlation of outcomes during repeated admissions. Age, sex and diagnosis (ICD-10 chapter), were added as covariates instead of carrying out separate analyses for each subgroup. To graphically plot changes in probability of death before and after the start of level 5 lockdown we calculated the probability of death each week, used a linear regression model equivalent to the second equation above, and plotted predicted probabilities of death over time.

As a sensitivity analysis, to assess possible confounding by seasonal effects on daily admissions and mortality we extended pre-lockdown data to the start of 2019, and added calendar month as covariates to each model.

We use a 5% significance level and 95% confidence intervals. Analysis was carried out with Stata 16.0.

Ethical Issues

Ethical approval was granted for AHRI to collect and use data from patients attending the clinics and hospital by the University of KwaZulu Natal Biomedical Research Ethics Committee: number and title BE290/16 “A longitudinal population-based platform for epidemiology and intervention research”. This was a secondary analysis on this anonymised data set with permission from AHRI, so further ethical approval was not required.

Patient and Public Involvement

There was no patient or public involvement in this study.

RESULTS

For peer review only - http://bmjopen.bmj.com/site/about/guidelines.xhtml

There were 6173 admissions by 5848 individuals to the study hospital from 1st January 2020 to 20th October 2020 (Table 1). 4.7% (n=291) of admissions resulted in death. 59.4% of all admissions were in 20-45 year-olds (n=3664), and more admissions were in females compared to males (77.6% vs. 22.4%, n=4788 vs. n=1385). The most common reasons for admission were maternal or neonatal conditions (32.0% n=3271), communicable diseases (CDs) (21.6% n=2203), and non-communicable diseases (NCDs) (14.2% n=1455). Ninety-two admissions were identified as having COVID-19, of which 66.3% occurred during level 3 lockdown (n=61) and 21.7% (n=20) during level 2 lockdown. LOWESS plots of crude time trends in admissions and mortality during 2019 and 2020 are shown graphically in Supplementary Figures 1-3.

Incidence rate ratios of numbers of daily admissions between each level of lockdown are shown in Table 2. Total daily admissions did not change significantly between levels, although they increased slightly from levels 4 to 3 (incidence rate ratio (IRR) 1.17, 95% confidence interval (CI) 1.06-1.28).

However, there were statistically significant changes among subgroups of patients. Daily admissions decreased significantly during the initial level 5 lockdown for infants (IRR 0.63, 95% CI 0.44-0.90), 1-5 year-olds (IRR 0.43, 95% CI 028-0.65) and for respiratory diagnoses (IRR 0.57, 95% CI 0.36-0.90). The most common reasons for admission in infants were neonatal conditions (42.6%), gastroenteritis (18.6%) and pneumonia (9.5%). In 1-5 year-olds they were injuries (32.1%), gastroenteritis (15.0%) and pneumonia (10.6%).

From level 4 to level 3, admissions increased significantly for men aged over 19 (IRR 1.50, 95% CI 1.17-1.92), 20-45 year-olds (IRR 1.19, 95% CI 1.05-1.34) and for respiratory diagnoses (IRR 4.26, 95% CI 2.36-7.70).

From level 2 to level 1, admissions decreased significantly for maternal and neonatal diagnoses (IRR 0.74, 95% CI 0.64-0.86), NCDs (IRR 0.76, 95% CI 0.60-0.96) and injuries (IRR 0.54, 95% CI 0.34-0.86). As a sensitivity analysis, admissions from the 1st of October onwards were excluded to test if this finding was due to data collection or upload delays in ICD-10 coding. The decrease seen in NCDs persisted but became non-significant (IRR 0.79, 95% CI 0.56-1.11). Maternal and neonatal admissions no longer decreased (IRR 1.00, 95% CI 0.83–1.22). Admissions for injuries continued to decrease (IRR 0.43, 95% CI 0.19-0.94). There were no statistically significant changes in these sub-groups during earlier stages of lockdown. Daily number of admissions in 6-19, 45-65, over 65 year olds, and for adult women and CDs did not change significantly at any stage of lockdown.

Linear regression models of these step changes produced similar results (Supplementary Table 1). Residuals of linear regression models were approximately normally distributed.

For peer review only - http://bmjopen.bmj.com/site/about/guidelines.xhtml

Poisson and linear regression models of continuous time trends before and after the start of level 5 lockdown also produced similar results, although some differences became non-significant (Supplementary Tables 2 and 3, Figures 1 and 2).

Among patients admitted to hospital, odds of death decreased substantially and significantly during level 5 lockdown compared to pre-lockdown (adjusted odds ratio (OR) 0.48; 95% CI 0.28-0.83), then increased from level 5 to level 4 (OR 1.94, 95% CI 1.01-3.71), did not change from level 4 to level 3 (OR 0.84, 95% CI 0.51-1.39), increased from level 3 to level 2 (OR 1.74, 95% CI 1.15-2.63), then did not change during level 1 (Table 3). Changes in odds of death were not affected by changes in age, sex or diagnosis, except that adjustment for diagnosis increased the odds ratios for levels 4 versus 5 and levels 2 versus 3 and made them statistically significant (Table 3, Supplementary Table 4). Figure 3 illustrates the large decrease in probability of death immediately after the start of level 5 lockdown, followed by an increase thereafter.

Inclusion of 2019 data and adjustment for calendar month did not substantially change any of these results.

For peer review only - http://bmjopen.bmj.com/site/about/guidelines.xhtml

Table 1. Numbers (%) of admissions to and deaths in Hlabisa hospital, rural KwaZulu Natal, South Africa, from January 2020-September 2020 according to sex, age and diagnosis.

	**Total** n (% of column total)	**Male** n (% of row total)	**Female** n (% of row total)	**Age <1** n (%of row total)	**Age 1-5** n (% of row total)	**Age 6-19** n (% of row total)	**Age 20-45** n (% of row total)	**Age 46-65** n (% of row total)	**Age>65** n (% of row total)
**Total** n (%)	6173 (100.0)*	1385 (22.4)	4788 (77.6)	334 (5.4)	242 (3.9)	951 (15.4)	3664 (59.4)	580 (9.4)	402(6.5)
**Maternal and neonatal** n (%)	3271 (32.0)	78 (2.4)	3193 (97.6)	145 (4.4)	7 (0.2)	662 (20.2)	2446 (74.8)	6 (0.2)	5 (0.2)
**Non-communicable diseases** n (%)	1455 (14.2)	568 (39.0)	887 (61.0)	59 (4.1)	70 (4.8)	109 (7.5)	517 (35.5)	386 (26.5)	314 (21.6)
**Communicable diseases** n (%)	2203 (21.6)	591 (26.8)	1612 (73.2)	129 (5.9)	103 (4.7)	110 (5.0)	1448 (65.7)	280 (12.7)	133 (6.04)
**Injuries** n (%)	496 (4.9)	302 (60.9)	194 (39.1)	19 (3.8)	83 (16.7)	104 (21.0)	225 (45.4)	46 (9.3)	19 (3.8)
**Respiratory** n (%)	323 (3.2)	151 (46.8)	172 (53.3)	42 (13.0)	34 (10.5)	15 (4.6)	92 (28.5)	69 (21.4)	71 (22.0)
**Other** n (%)	2786 (27.3)	556 (20.0)	2230 (80.0)	194 (7.0)	124 (4.5)	672 (35.5)	1531 (55.0)	158 (5.7)	107 (3.8)
**Deaths** n (%)	291 (4.7)	139 (47.8)	152 (52.2)	18 (6.2)	2 (0.7)	7 (2.4)	111 (38.14)	80 (27.5)	73 (25.1)

*Primary and secondary diagnosis used for each admission, therefore total of column % exceed 100%.

For peer review only

Table 2: Step changes in daily incidence of admissions at each level of lockdown: Poisson regression models adjusted for day of week

	Pre-lockdown admission daily incidence rate (95% CI)			IRR at level 5 vs 4	95% CI		P value	IRR at level 4 vs 5	95% CI		P value	IRR at level 3 vs 4	95% CI		P value	IRR at level 2 vs 3	95% CI		P value	IRR at level 1 vs 2	95% CI		P value
Total	17.08	15.73	18.55	0.96	0.88	1.05	0.41	0.99	0.88	1.10	0.81	1.17	1.06	1.28	0.001	1.02	0.94	1.11	0.67	1.02	0.94	1.11	0.67
Male >19	2.59	2.11	3.19	0.95	0.75	1.21	0.70	0.93	0.69	1.25	0.61	1.50	1.17	1.92	0.001	0.98	0.80	1.21	0.87	0.93	0.72	1.21	0.59
Female>19	8.96	8.02	10.00	1.05	0.93	1.18	0.43	1.03	0.89	1.18	0.71	1.11	0.99	1.25	0.08	1.04	0.93	1.16	0.49	0.92	0.80	1.05	0.20
Age <1	1.46	1.04	2.03	0.63	0.44	0.90	0.01	0.60	0.35	1.02	0.06	1.52	0.93	2.49	0.09	0.75	0.48	1.18	0.21	1.17	0.67	2.02	0.58
Age 1-5	1.07	0.99	1.17	0.43	0.28	0.65	<0.001	0.88	0.47	1.66	0.70	1.50	0.88	2.55	0.14	0.57	0.33	0.98	0.04	1.75	0.94	3.28	0.08
Age 6-19*	3.11	2.56	3.79	0.99	0.80	1.24	0.95	1.05	0.80	1.37	0.74	0.98	0.78	1.24	0.89	1.17	0.95	1.45	0.14	0.76	0.58	0.99	0.05
Age 20-45	9.13	8.18	10.18	1.08	0.96	1.21	0.20	0.95	0.83	1.10	0.50	1.19	1.05	1.34	0.01	1.05	0.95	1.17	0.34	0.90	0.79	1.03	0.12
Age 46-65	1.29	0.96	1.72	0.89	0.65	1.21	0.44	1.24	0.86	1.78	0.26	1.22	0.91	1.64	0.18	0.77	0.58	1.03	0.08	1.16	0.82	1.65	0.40
Age >65	1.14	0.83	1.57	0.80	0.56	1.17	0.25	1.29	0.83	2.01	0.26	1.04	0.73	1.48	0.83	1.18	0.86	1.63	0.31	0.83	0.55	1.23	0.35
Maternal and neonatal	8.28	7.38	0.00	1.09	0.97	1.23	0.17	1.04	0.90	1.21	0.56	1.03	0.91	1.16	0.68	1.08	0.97	1.21	0.17	0.74	0.64	0.86	<0.001
NCDs	3.84	3.23	4.57	0.90	0.75	1.08	0.27	1.15	0.92	1.43	0.23	1.07	0.89	1.29	0.46	0.90	0.76	1.08	0.27	0.76	0.60	0.96	0.02
CDs	6.34	5.51	0.00	0.88	0.77	1.02	0.10	0.95	0.79	1.15	0.62	1.14	0.98	1.34	0.10	0.91	0.78	1.05	0.20	0.86	0.71	1.04	0.11
Injuries	2.56	2.01	3.00	0.80	0.59	1.08	0.14	0.83	0.56	1.24	0.37	1.34	0.95	1.88	0.10	0.88	0.64	1.20	0.42	0.54	0.34	0.86	0.01
Respiratory	0.70	0.47	1.06	0.57	0.36	0.90	0.02	0.59	0.30	1.20	0.15	4.26	2.36	7.70	<0.001	0.58	0.40	0.85	0.01	0.89	0.53	1.48	0.65

Table 3: Step changes in odds of death at Hlabisa hospital at each level of lockdown: logistic regression models*

	Pre-lockdown odds (95% CI)			OR level 5 vs pre	95% CI		P value	OR level 4 vs 5	95% CI		P value	OR level 3 vs 4	95% CI		P value	OR level 2 vs 3	95% CI		P value	OR level 1 vs 2	95% CI		P value
Unadjusted	0.05	0.04	0.07	0.48	0.28	0.80	<0.001	1.81	0.97	3.38	0.06	0.98	0.62	1.54	0.92	1.40	0.96	2.04	0.08	0.98	0.62	1.53	0.92
Adjusted for day of week, Sex and Age	0.02	0.02	0.04	0.47	0.28	0.80	<0.001	1.75	0.93	3.28	0.08	0.96	0.59	1.54	0.85	1.42	0.95	2.12	0.09	0.97	0.60	1.57	0.91
Adjusted for day of week, sex, age and diagnosis	0.04	0.03	0.07	0.48	0.28	0.83	0.01	1.94	1.01	3.71	0.05	0.84	0.51	1.39	0.51	1.74	1.15	2.63	0.01	1.00	0.59	1.71	0.99

OR Odds ratio

* analysis at level of admission, with robust adjustment for intra-person correlation of outcome

For peer review only - http://bmjopen.bmj.com/site/about/guidelines.xhtml

DISCUSSION

In this analysis of hospital admission and hospital mortality in a mostly rural South African setting, national lockdown was not associated with reductions in pooled daily admissions for all causes and all ages. Total admissions, and of adult men, increased from level 4 to level 3. Numbers of admissions in adult women, over 45 year olds, 6-19 year olds, and for communicable diseases, were mostly unchanged after lockdown. However, admissions for children under five fell sharply during level 5 lockdown and remained low. During the final level 1 lockdown, admissions for maternal and neonatal conditions, non-communicable diseases and injuries dropped significantly compared to level 2, despite no significant changes during earlier levels of lockdown. Risk of in-hospital mortality halved during level 5 lockdown then increased to pre-lockdown rates. These observed changes could be due to changes in the health of the local population, or to lockdown restrictions, or both. However, the magnitude of changes over a relatively short period and the small number of reported COVID-19 admissions, suggest that changes in access to hospital care are likely to have been influential. Reduced access to hospital during lockdown could be due to a combination of factors including mandatory restrictions on movement, fear of being infected, and inability to pay for transport due to lost income (hospital care is free to users).

Our findings that overall hospital admissions did not fall, but that admissions of under five year olds decreased, were consistent with findings of Siedner and colleagues on primary care visitations in the same population[24]. However, in the present study under-five hospital admissions did not increase to normal after level 5, unlike primary care visits which did[24]. Reasons for reduced admissions in under-fives could include reduction in circulating viral illnesses following school closures, or reduction in injuries as children spend more time at home during the strict lockdown measures of level 5. More concerningly, it could signify that unwell children were not being taken to hospital by caregivers for acute illnesses such as pneumonia or influenza. This could explain the concurrent drop in respiratory admissions – particularly given that a large proportion of respiratory admissions were in children and most were due to pneumonia. Reduced access to hospital during level 5 lockdown thus appears to affect children more than other sub-groups, highlighting the vulnerability of children and their dependence on carers, who might have been concerned to protect their young children and themselves from COVID-19 infection.

Similar reductions in paediatric admissions for respiratory disease were reported during the early pandemic in China , and decreased emergency department attendance was reported in Victoria, Australia[27,28]. Modelling by Roberton and colleagues predicted that COVID-19 could cause at least 253,500 additional child deaths in low and middle income countries,

For peer review only - http://bmjopen.bmj.com/site/about/guidelines.xhtml

41% of which would be attributable to reduced acute care for childhood illnesses, such as antibiotic treatment of pneumonia[17].

These results suggest that admissions for over 45 years olds, communicable diseases and injuries remained stable despite the significant restrictions during lockdown. This is unlike other regions in South Africa, which saw reductions in surgical admissions in North West Province, and prenatal anti-retroviral pre-exposure prophylaxis visits in the Western Cape[29,30].

The KwaZulu-Natal provincial health department reported that by 11th April the cumulative incidence of COVID-19 in uMkhanyakude district was 21, with no deaths[31]. By 5th of August the cumulative incidence had risen to 1513 cases, accounting for 1.7% of cases in the province. The daily incidence rate was less than 50 cases per 100,000 population[32]. This was despite a peak in incidence of cases nationally in July[23]. Respiratory admissions in this study population (which excluded COVID-19) peaked during level 3, which coincides with the peak in COVID-19 incidence in KwaZulu-Natal. It is possible that undiagnosed COVID-19 presenting as pneumonia could account for some of this increase. The majority of admissions with a primary diagnosis of COVID-19 also occurred during level 3 at Hlabisa, and the combination of both diagnosed and undiagnosed COVID-19 may also partly account for the increase in admissions in men and 20-45 year olds during level 3 lockdown.

A possible explanation for the increase during level 3 could be migration into the area, if urban workers moved back to their rural homes when transport restrictions eased during level 3 lockdown[33]. However, Siedner and colleagues interrogated local longitudinal primary care attendance data and did not find evidence of substantial migration[24]. The temporary lifting of the ban on alcohol sales at the beginning of level 2 lockdown[20] could have led to an increase in alcohol-related admissions in these sub-groups, but admission for injuries or NCDs, which are often alcohol-related, did not change significantly during level 2 lockdown. Admissions for NCDs and injuries also did not decrease significantly at the beginning of the lockdown when the alcohol ban commenced, as seen in the Western Cape and Cape Town [30,34].

A major finding of this study is the large reduction in all-cause mortality during level 5 lockdown, despite resilience in admissions numbers overall. This supports our hypothesis that the sickest people may have been disproportionately unable to access hospital care as needed and died at home as opposed to hospital, or presented late to hospital resulting in increasing mortality from level 4 onwards. Reductions in numbers of admissions in the sickest may have been offset by the observed increases in admissions of younger, healthier adults. The South African Medical Research Council reported 7729 excess deaths in

For peer review only - http://bmjopen.bmj.com/site/about/guidelines.xhtml

KwaZulu-Natal from 6th May to 10th November 2020, compared to the same dates during previous years, which was much more than the number of COVID-19 cases reported[35]. These excess deaths were probably due both to undiagnosed COVID-19 and to undertreatment of other life-threatening conditions[35,36]. It is thus likely that reduced utilisation by the sickest may have resulted in preventable death outside of hospital. Further analysis of local demographic data on rates and place of death during lockdown may elucidate this finding.

Limitations of this study include only analysing admissions up till 20th October 2020, before the pandemic had resolved globally and the second waves of COVID-19 cases were beginning[37]. Therefore, the impact of the epidemic on long-term healthcare utilisation or hospital mortality cannot yet be assessed in this study. Our analysis is restricted by a relatively small data set from one district hospital, which may have limited the power of our study to detect effects of lockdown, particularly in sub-group analysis and the generalisability to other secondary care settings. These hospital data is unable to account for changes in local epidemiology and need for health services, or for societal changes at the time which may have influenced the results. While there were no other known significant events during lockdown in the region likely to have influenced utilisation of hospital care, we cannot exclude other influences such as weather or socio-cultural factors.

Further work should focus on the impact of COVID-19 on access to children's health services. They appear to have a lower risk of acquiring and suffering from severe COVID-19[38–40], but the implications of strict lockdowns on young children's access and utilisation to healthcare for acute illnesses is concerning. Also important is access to emergency transport to hospital for people with life-threatening conditions.

For peer review only

REFERENCES

1 Kinross P, Suetens C, Gomes Dias J, *et al.* Rapidly increasing cumulative incidence of coronavirus disease (COVID-19) in the European Union/European Economic Area and the United Kingdom, 1 January to 15 March 2020. *Eurosurveillance* 2020;**25**:2000285. doi:10.2807/1560-7917.ES.2020.25.11.2000285

2 Italy's Health Care System Groans Under Coronavirus — a Warning to the World - The New York Times. https://www.nytimes.com/2020/03/12/world/europe/12italy-coronavirus-health-care.html (accessed 10 Nov 2020).

3 Dunn P, Allen L, Cameron G, *et al.* COVID-19 policy tracker | The Health Foundation. 2020.https://www.health.org.uk/news-and-comment/charts-and-infographics/covid-19-policy-tracker (accessed 10 Nov 2020).

4 World Health Organization. Statement on the third meeting of the International Health Regulations (2005) Emergency Committee regarding the outbreak of coronavirus disease (COVID-19). 1 May 2020. 2020. https://www.who.int/news/item/01-05-2020-statement-on-the-third-meeting-of-the-international-health-regulations-(2005)-emergency-committee-regarding-the-outbreak-of-coronavirus-disease-(covid-19) (accessed 28 Oct 2020).

5 Haffajee F. Post-Covid economy resembles a post-war landscape as joblessness climbs to highest ever. 2020.https://www.dailymaverick.co.za/article/2020-09-30-post-covid-economy-resembles-a-post-war-landscape-as-joblessness-climbs-to-highest-ever/ (accessed 10 Nov 2020).

6 Ewing J. Europe Risks a New Economic Downturn as Lockdowns Return. https://www.nytimes.com/2020/10/30/business/european-economy.html (accessed 10 Nov 2020).

7 United Nations Children's Fund. Education on hold | UNICEF Latin America and Caribbean. 2020. https://www.unicef.org/lac/en/reports/education-on-hold (accessed 10 Nov 2020).

8 Bulled N, Singer M. In the shadow of HIV & TB: A commentary on the COVID epidemic in South Africa. *Glob Public Health* 2020;**15**:1231–43. doi:10.1080/17441692.2020.1775275

9 Jiang H, Zhou Y, Tang W. Maintaining HIV care during the COVID-19 pandemic. *Lancet HIV* 2020;**7**:e308–9. doi:https://doi.org/10.1016/S2352-3018(20)30105-3

10 United Nations Family Planning Association. Interim Technical Note Impact of the COVID-19 Pandemic on Family Planning and Ending Gender-based Violence, Female Genital Mutilation and Child Marriage.

11 World Health Organization, United Nations Children's Fund. Protect the Progress: Rise, Refocus, Recover. 2020 Progress Report on the Every Women Every Child Global Strategy for Women's, Children's and Adolescents' Health (2016-2030). 2020. https://protect.everywomaneverychild.org/assets/img/2020-Progress-Report-on-the-EWEC-Global-Strategy-Final.pdf (accessed 29 Sep 2020).

12 Nogueira PJ, De Araújo Nobre M, Nicola PJ, *et al.* Excess Mortality Estimation During the COVID-19 Pandemic: Preliminary Data from Portugal. *Acta Med Port* 2020;**33**:376. doi:10.20344/amp.13928

13 Kontis V, Bennett JE, Rashid T, *et al.* Magnitude, demographics and dynamics of the effect of the first wave of the COVID-19 pandemic on all-cause mortality in 21 industrialized countries. *Nat Med* Published Online First: 2020. doi:10.1038/s41591-

For peer review only - http://bmjopen.bmj.com/site/about/guidelines.xhtml

020-1112-0

14 Abbas K, Procter SR, van Zandvoort K, *et al.* Routine childhood immunisation during the COVID-19 pandemic in Africa: a benefit-risk analysis of health benefits versus excess risk of SARS-CoV-2 infection. *Lancet Glob Heal* 2020;**0**. doi:10.1016/S2214-109X(20)30308-9

15 Hogan AB, Jewell BL, Sherrard-Smith E, *et al.* Potential impact of the COVID-19 pandemic on HIV, tuberculosis, and malaria in low-income and middle-income countries: a modelling study. *Lancet Glob Heal* 2020;**0**. doi:10.1016/S2214-109X(20)30288-6

16 Jewell BL, Mudimu E, Stover J, *et al.* Potential effects of disruption to HIV programmes in sub-Saharan Africa caused by COVID-19: results from multiple mathematical models. *Lancet HIV* 2020;**7**:e629–40. doi:10.1016/S2352-3018(20)30211-3

17 Roberton T, Carter ED, Chou VB, *et al.* Early estimates of the indirect effects of the COVID-19 pandemic on maternal and child mortality in low-income and middle-income countries: a modelling study. *Lancet Glob Heal* 2020;**8**:e901–8. doi:10.1016/S2214-109X(20)30229-1

18 South Africa Department of Health. Disaster Management Act 2002 (Act No.57 of 2002) Alert level 5 during Coronavirus COVID-19 lockdown. Government Gazette No 43148:398. 25 March 2020.

19 South Africa Department of Health. Disaster Management Act 2002 (Act No.57 of 2002) Alert level 4 during Coronavirus COVID-19 lockdown. Government Gazette No 43258:480. 29 April 2020.

20 South Africa Department of Health. Disaster Management Act 2002 (Act No.57 of 2002) Alert level 3 during Coronavirus COVID-19 lockdown. Government Gazette No 43364:608. 28 May 2020.

21 South Africa Department of Health. Disaster Management Act 2002 (Act No.57 of 2002) Alert level 2 during Coronavirus COVID-19 lockdown. Government Gazette No 43620:891. 17 August 2020.

22 South Africa Department of Health. Disaster Management Act 2002 (Act No.57 of 2002) Alert level 1 during Coronavirus COVID-19 lockdown. Government Gazette No 43725:999. 18 September 2020.

23 National Institute for Communicable Diseases. COVID-19 WEEKLY EPIDEMIOLOGY BRIEF South Africa Week 42 2020. 2020.

24 Siedner MJ, Kraemer JD, Meyer MJ, *et al.* Access to primary healthcare during lockdown measures for COVID-19 in rural South Africa: an interrupted time series analysis. *BMJ Open* 2020;**10**. doi:10.1136/bmjopen-2020-043763

25 Massyn N, Day C, Ndlovu N, *et al.* District Health Barometer: District Health Profiles 2018/19. 2020.

26 World Health Organization. ICD-10 : international statistical classification of diseases and related health problems : tenth revision, 2nd ed. 2004.https://icd.who.int/browse10/2019/en (accessed 25 Oct 2020).

27 Cheek JA, Craig SS, West A, *et al.* Emergency department utilisation by vulnerable paediatric populations during the COVID-19 pandemic. *Emerg Med Australas* 2020;**32**:870–1. doi:10.1111/1742-6723.13598

For peer review only - http://bmjopen.bmj.com/site/about/guidelines.xhtml

28 Li H, Yu G, Duan H, *et al.* Changes in Children's Healthcare Visits During Coronavirus Disease-2019 Pandemic in Hangzhou, China. *J Pediatr* 2020;**224**:146. doi:10.1016/J.JPEDS.2020.05.013

29 Davey DLJ, Bekker L-G, Mashele N, *et al.* PrEP retention and prescriptions for pregnant women during COVID-19 lockdown in South Africa. *Lancet HIV* Published Online First: August 2020. doi:10.1016/S2352-3018(20)30226-5

30 Moustakis J, Piperidis AA, Ogunrombi AB. The effect of COVID-19 on essential surgical admissions in South Africa: A retrospective observational analysis of admissions before and during lockdown at a tertiary healthcare complex. *South African Med J* 2020;**110**:910. doi:10.7196/SAMJ.2020.v110i9.15025

31 KwaZulu-Natal Department of Health, National Institute for Communicable Diseases. KwaZulu-Natal Department of Health COVID-19 Situation report :12 April 2020. 2020.

32 KwaZulu-Natal Department of Health, National Institute for Communicable Diseases. KwaZulu-Natal Department of Health COVID-19 Situational report: 5 August 2020. 2020.

33 Bank L. Covid-19 reveals migration links in South Africa's human economy. 2020.https://www.dailymaverick.co.za/article/2020-05-17-covid-19-reveals-migration-links-in-south-africas-human-economy/ (accessed 27 Oct 2020).

34 Reuter H, Jenkins LS, De Jong M, *et al.* Prohibiting alcohol sales during the coronavirus disease 2019 pandemic has positive effects on health services in South Africa. *African J Prim Heal care Fam Med* 2020;**12**:e1–4. doi:10.4102/phcfm.v12i1.2528

35 South African Medical Research Council. Report on Weekly Deaths in South Africa. 2020.https://www.samrc.ac.za/reports/report-weekly-deaths-south-africa (accessed 19 Oct 2020).

36 Dyer O. Covid-19: Excess deaths point to hidden toll in South Africa as cases surge. *BMJ* 2020;**370**:m3038. doi:https://dx.doi.org/10.1136/bmj.m3038

37 Looi M-K. Covid-19: Is a second wave hitting Europe? *BMJ* 2020;**371**:m4113. doi:10.1136/bmj.m4113

38 Viner RM, Mytton OT, Bonell C, *et al.* Susceptibility to SARS-CoV-2 Infection Among Children and Adolescents Compared With Adults: A Systematic Review and Meta-analysis. *JAMA Pediatr* Published Online First: 25 September 2020. doi:10.1001/jamapediatrics.2020.4573

39 Christophers B, Gallo Marin B, Oliva R, *et al.* Trends in clinical presentation of children with COVID-19: a systematic review of individual participant data. *Pediatr Res* Published Online First: 2020. doi:10.1038/s41390-020-01161-3

40 Ludvigsson JF. Systematic review of COVID-19 in children shows milder cases and a better prognosis than adults. *Acta Paediatr* 2020;**109**:1088–95. doi:10.1111/apa.15270

For peer review only - http://bmjopen.bmj.com/site/about/guidelines.xhtml

FUNDING STATEMENT

The Africa Health Research Institute is funded by Wellcome Trust award 201433/Z/16/Z.

COMPETING INTERESTS

None declared

AUTHOR CONTRIBUTIONS

MB, MJS, JS and KH conceived the study aims and hypothesis.

KH and DG provided AHRI Hospital Information System data.

AM and MB designed the study and conducted the statistical analysis, with advice from MJS.

AM drafted the manuscript and prepared the tables, figures and final version for publication with substantial amendment and oversight from MB.

All authors discussed the results, critically revised the study and approved the final version of the manuscript.

ACKNOWLEDGMENTS

We thank the research assistants, nurses and staff at Hlabisa hospital and AHRI who contributed towards data collection. Thanks to Anita Edwards for proof-reading and support in preparing the manuscript for publication.

DATA SHARING

Statistical coding available on reasonable request from corresponding author. AHRI Hospital Information System data are available on request here: https://data.ahri.org/index.php/home.

For peer review only - http://bmjopen.bmj.com/site/about/guidelines.xhtml

LEGENDS

Table 1. Numbers (%) of admissions to and deaths in Hlabisa hospital, rural KwaZulu Natal, South Africa, from January 2020-September 2020 according to sex, age and diagnosis.

*Primary and secondary diagnosis used for each admission, therefore total of column % exceed 100%.

Table 2: Step changes in daily incidence of admissions at each level of lockdown: Poisson regression models adjusted for day of week

Table 3: Step changes in odds of death at Hlabisa hospital at each level of lockdown: logistic regression models*

* analysis at level of admission, with robust adjustment for intra-person correlation of outcome

OR: odds ratio

Figure 1: Admissions to Hlabisa hospital from January 2020 to October 2020 for all reasons, by Sex and Age. Scatter plot represents number of admissions per day. Black line represents fitted linear regression model with adjustment for day of week, before and after lockdown. Dashed lines represent confidence intervals of the fitted model. Red line represents the beginning of lockdown level 5.

Figure 2: Admissions to Hlabisa hospital from January 2020 to October 2020, by discharge diagnosis. Scatter plot represents number of admissions per day. Black line represents fitted linear regression model with adjustment for day of week, before and after lockdown. Dashed lines represent confidence intervals of the fitted model. Red line represents the beginning of lockdown level 5.

Figure 3: Probability of death among all admissions each week: observed and predicted by interrupted times series linear regression model. Scatter plot represents probability of death each week. Black line represents fitted linear regression model with adjustment for day of week, before and after lockdown. Dashed lines represent confidence intervals of the fitted model. Red line represents the beginning of lockdown level 5.

For peer review only

Figure 1: Admissions to Hlabisa hospital from January 2020 to October 2020 for all reasons, by Sex and Age. Scatter plot represents number of admissions per day. Black line represents fitted linear regression model with adjustment for day of week, before and after lockdown. Dashed lines represent confidence intervals of the fitted model. Red line represents the beginning of lockdown level 5.

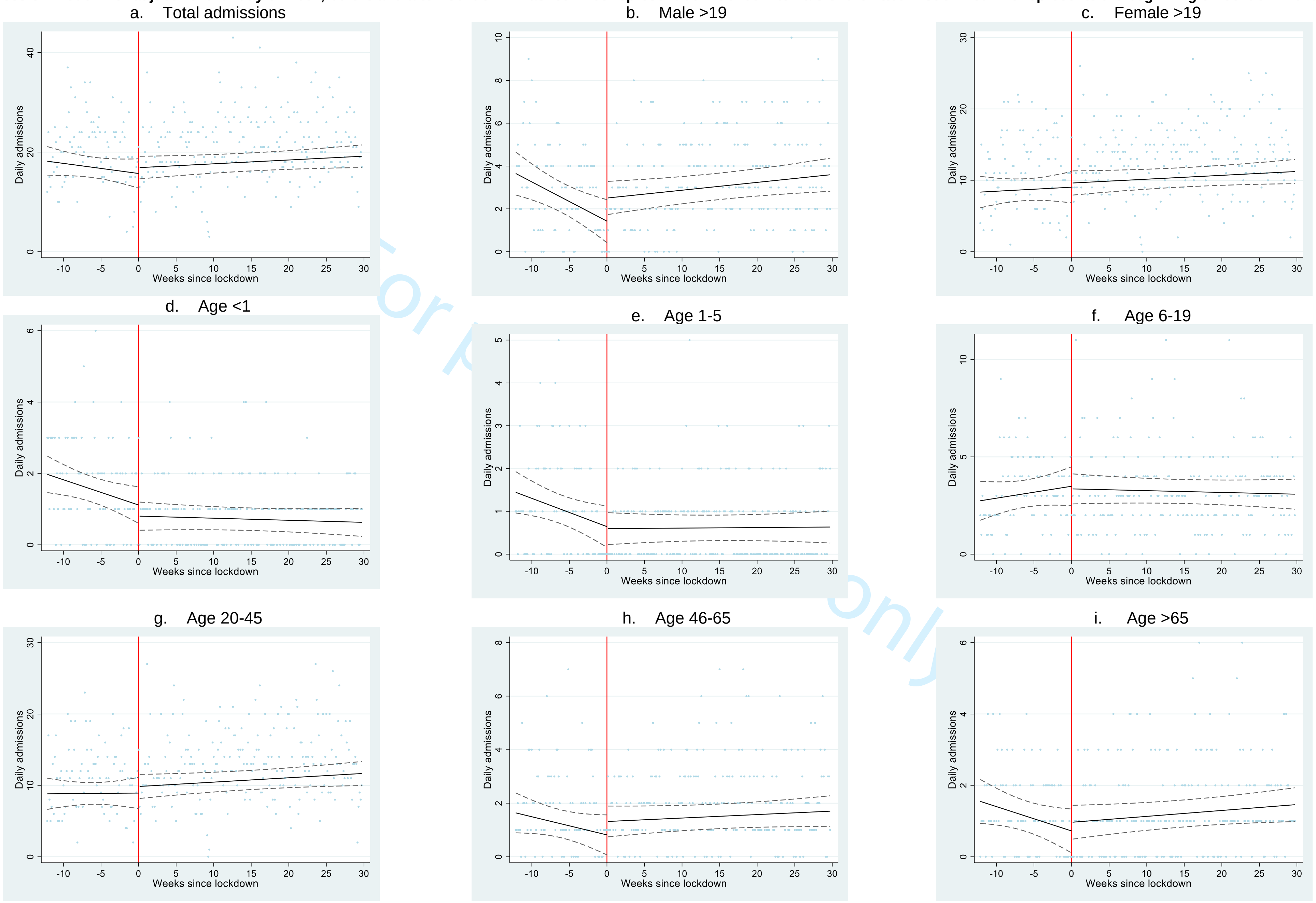

Figure 2

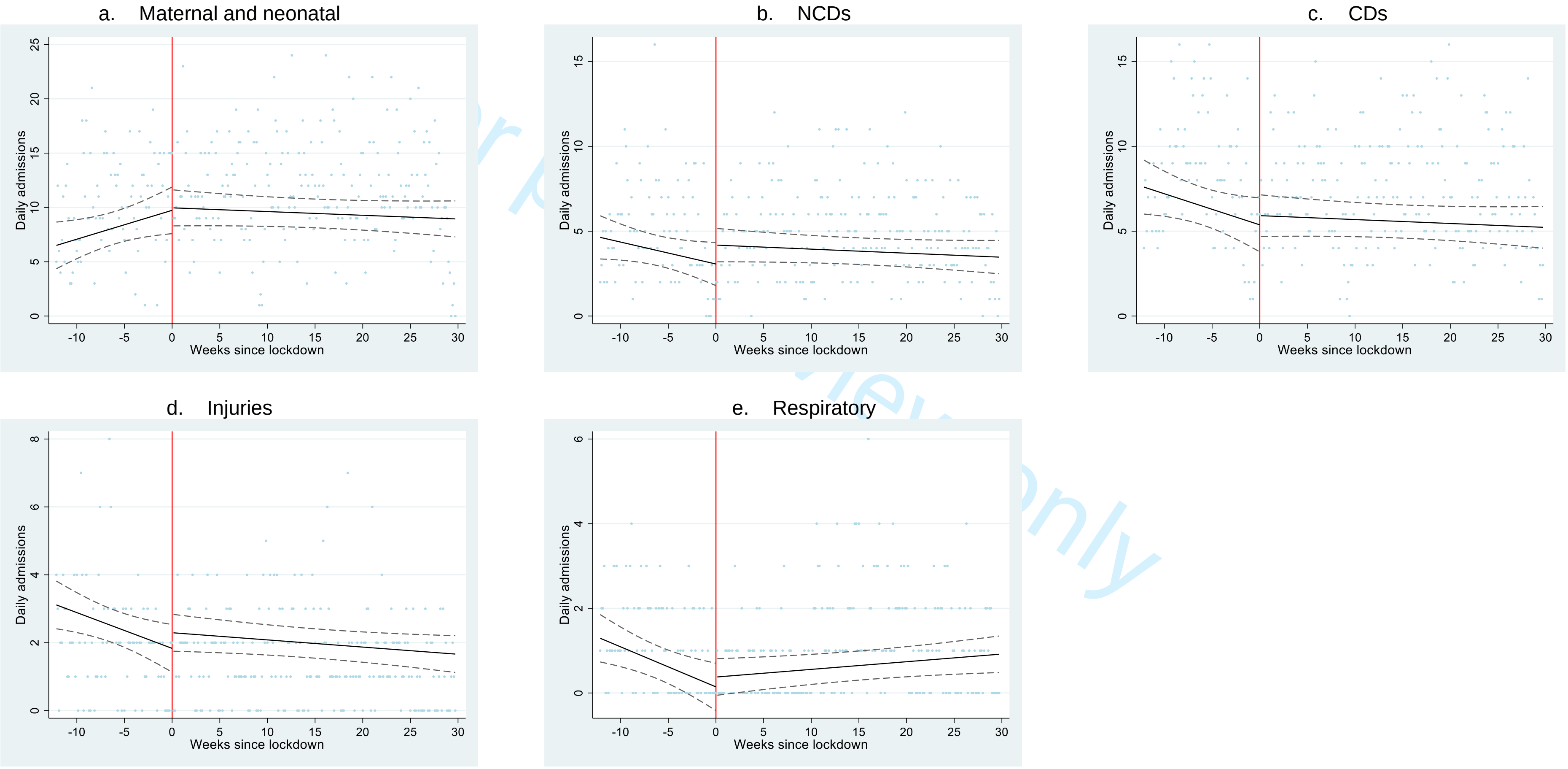
a. Maternal and neonatal
b. NCDs
c. CDs
d. Injuries
e. Respiratory
Daily admissions
Weeks since lockdown

Figure 3. Probability of death among all admissions each week: observed and predicted by interrupted times series linear regression model. Black line represents fitted linear regression model with adjustment for day of week, before and after lockdown. Dashed lines represent confidence intervals of the fitted model. Red line represents the beginning of lockdown level 5.

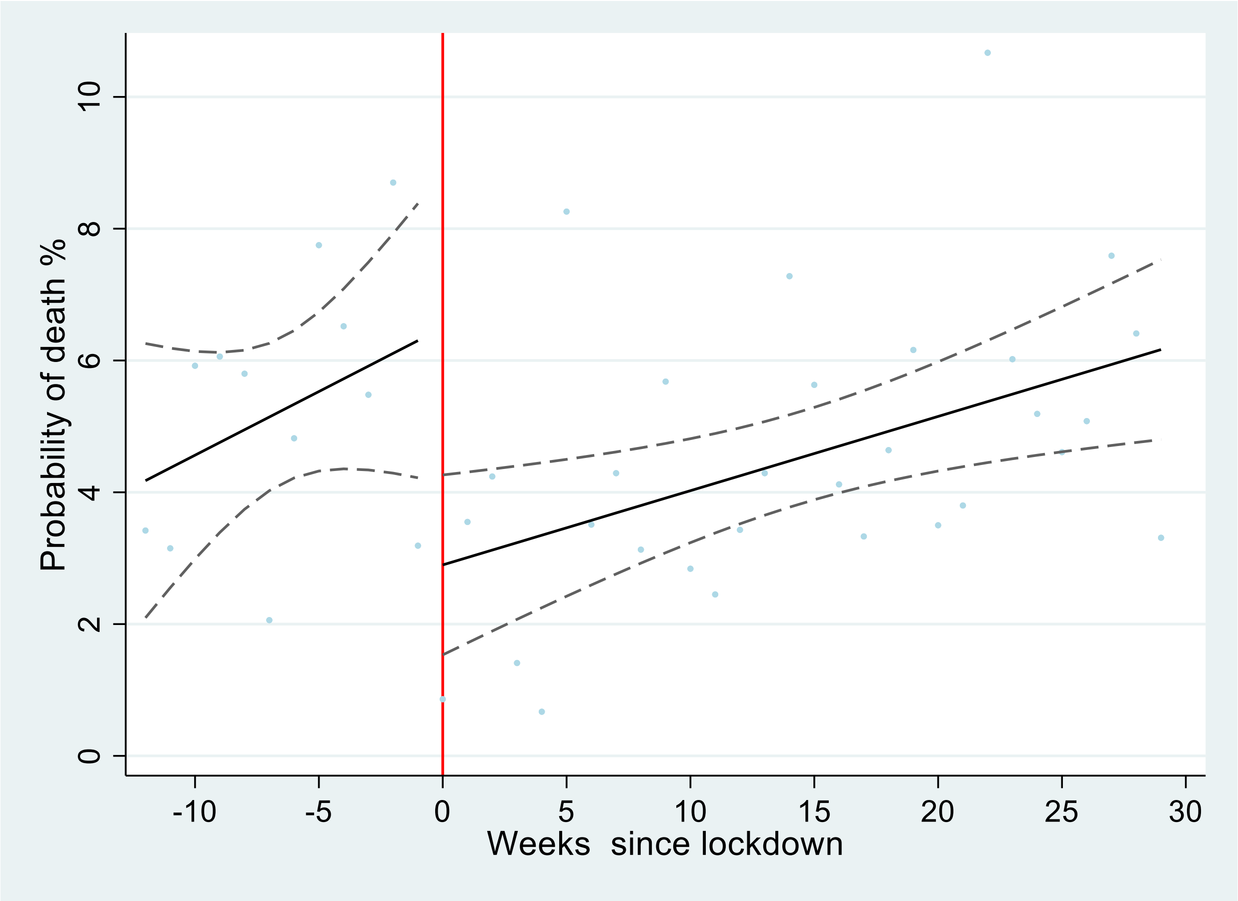

Supplementary Table 1: Changes in mean daily admissions to Hlabisa hospital at each stage of lockdown: linear regression models adjusted for day of week

	Pre-lockdown average daily admissions (95% CI)			Change at level 5	95% CI		P value	Change at level 4	95% CI		P value	Change at level 3	95% CI		P value	Change at level 2	95% CI		P value	Change at level 1	95% CI		P value
All admissions	16.96	14.93	19.00	-0.74	-3.02	1.53	0.52	-0.23	-3.03	2.57	0.87	3.18	0.77	5.59	0.01	0.40	-1.94	2.73	0.74	-1.88	-4.72	0.97	0.20
Male >19	2.55	1.85	3.26	-0.13	-0.92	0.66	0.74	-0.20	-1.17	0.77	0.68	1.27	0.44	2.11	<0.001	-0.07	-0.88	0.74	0.87	-0.25	-1.24	0.73	0.61
Female >19	8.70	7.17	10.23	0.54	-1.17	2.26	0.53	0.33	-1.78	2.44	0.76	1.36	-0.46	3.17	0.14	0.51	-1.25	2.27	0.57	-1.17	-3.31	0.97	0.28
Age <1	1.55	1.19	1.91	-0.64	-1.04	-0.23	<0.001	-0.44	-0.93	0.06	0.08	0.34	-0.09	0.76	0.12	-0.25	-0.66	0.17	0.24	0.12	-0.38	0.62	0.64
Age 1-5	1.05	0.71	1.39	-0.50	-0.88	-0.12	0.01	-0.07	-0.53	0.40	0.78	0.27	-0.13	0.67	0.19	-0.36	-0.74	0.03	0.07	0.35	-0.13	0.82	0.15
Age 6-19	3.11	2.41	3.81	-0.02	-0.80	0.76	0.96	0.14	-0.82	1.11	0.77	-0.05	-0.88	0.77	0.90	0.56	-0.25	1.36	0.17	-0.92	-1.90	0.06	0.07
Age 20-45	8.89	7.37	10.40	0.87	-0.82	2.56	0.31	-0.55	-2.63	1.53	0.60	2.14	0.34	3.93	0.02	0.72	-1.01	2.46	0.41	-1.44	-3.55	0.68	0.18
Age 46-65	1.23	0.71	1.75	-0.20	-0.78	0.38	0.50	0.37	-0.34	1.09	0.31	0.43	-0.18	1.05	0.17	-0.54	-1.13	0.06	0.08	0.30	-0.43	1.03	0.42
Age >65	1.14	0.70	1.57	-0.26	-0.74	0.23	0.30	0.31	-0.29	0.90	0.31	0.06	-0.46	0.57	0.83	0.26	-0.24	0.75	0.31	-0.29	-0.90	0.31	0.35
Maternal and neonatal	8.10	6.61	9.60	0.90	-0.77	2.57	0.29	0.47	-1.58	2.53	0.65	0.31	-1.46	2.08	0.73	0.99	-0.72	2.70	0.26	-3.33	-5.41	-1.24	0.002
NCDs	3.84	2.96	4.72	-0.48	-1.47	0.50	0.34	0.65	-0.56	1.87	0.29	0.37	-0.67	1.41	0.49	-0.53	-1.54	0.48	0.30	-1.23	-2.46	-0.001	0.05
CDs	6.49	5.38	7.60	-0.94	-2.18	0.30	0.14	-0.32	-1.84	1.21	0.68	0.97	-0.34	2.29	0.15	-0.73	-2.00	0.55	0.26	-1.04	-2.59	0.51	0.19
Injuries	2.47	1.98	2.96	-0.41	-0.96	0.14	0.15	-0.27	-0.94	0.41	0.44	0.46	-0.13	1.04	0.13	-0.22	-0.78	0.35	0.45	-0.74	-1.43	-0.06	0.03
Respiratory	0.73	0.35	1.10	-0.49	-0.91	-0.08	0.02	-0.26	-0.77	0.25	0.32	1.27	0.83	1.71	<0.001	-0.70	-1.12	-0.27	0.002	-0.11	-0.63	0.41	0.68

NCDs: non-communicable disease

CDs: communicable diseases

CI: confidence interval

For peer review only

Supplementary Table 2: Time trends and step changes in daily hospital admissions before and after start of level 5 lockdown: Poisson regression models adjusted for day of week

	Pre-lockdown daily incidence rate	95% Confidence Interval		IRR pre-lockdown (per week)	95% Confidence Interval		P value	IRR at lockdown	Confidence Interval		P value	IRR post-lockdown (per week)	95% Confidence Interval		P value	P value of interaction term
All admissions	16.06	14.30	18.03	0.990	0.977	1.003	0.14	1.06	0.95	1.19	0.31	1.004	1.000	1.007	0.04	0.05
Male >19	1.71	1.24	2.36	0.94	0.91	0.97	<0.001	1.50	1.10	2.06	0.01	1.011	1.002	1.020	0.01	<0.001
Female>19	9.23	7.92	10.76	1.01	0.99	1.02	0.57	1.05	0.91	1.21	0.51	1.004	1.000	1.009	0.06	0.92
Age <1	1.12	0.71	1.75	0.96	0.92	1.004	0.08	0.74	0.48	1.16	0.19	0.99	0.98	1.01	0.45	0.16
Age 1-5	0.69	0.40	1.19	0.94	0.89	0.998	0.04	0.88	0.51	1.52	0.65	1.00	0.98	1.02	0.85	0.05
Age 6-19	3.50	2.65	4.62	1.02	0.99	1.05	0.26	0.96	0.73	1.26	0.76	1.00	0.99	1.01	0.53	0.21
Age 20-45	9.17	7.86	10.69	1.00	0.98	1.02	0.91	1.08	0.93	1.25	0.29	1.005	1.0003	1.01	0.04	0.69
Age 46-65	1.01	0.67	1.52	0.96	0.92	1.01	0.10	1.34	0.91	1.96	0.14	1.006	0.995	1.018	0.26	0.06
Age >65	0.82	0.51	1.32	0.95	0.90	1.0003	0.05	1.23	0.77	1.96	0.39	1.012	0.998	1.026	0.08	0.02
Maternal and neonatal	9.67	8.27	11.30	1.03	1.01	1.05	0.01	1.01	0.87	1.18	0.86	0.997	0.992	1.002	0.21	0.003
NCDs	3.27	2.57	4.18	0.97	0.95	1.001	0.06	1.26	0.998	1.58	0.05	0.995	0.988	1.002	0.19	0.14
CDs	5.52	4.55	6.00	0.98	0.96	0.998	0.04	1.07	0.89	1.28	0.47	0.997	0.991	1.003	0.29	0.08
Injuries	1.83	1.26	2.00	0.95	0.91	0.99	0.02	1.29	0.88	1.89	0.19	0.986	0.974	0.999	0.04	0.09
Respiratory	0.40	0.23	0.71	0.92	0.87	0.97	0.004	1.25	0.73	2.12	0.41	1.02	1.002	1.03	0.03	0.001

NCDs: non-communicable disease

CDs: communicable diseases

For peer review only

Supplementary Table 3. Time trends and step changes in daily hospital admissions before and after start of level 5 lockdown: linear regression models adjusted for day of week

	Average admissions day before lockdown	95% Confidence Interval		Change in daily admissions/week pre-lockdown	95% Confidence Interval		P value	Change in daily admissions at lockdown	95% Confidence Interval		P value	Change in daily admissions/week post-lockdown	95% Confidence Interval		P value	P value of interaction term
All admissions	15.71	12.75	18.66	-0.20	-0.55	0.15	0.26	1.17	-1.76	4.10	0.43	0.08	-0.02	0.17	0.10	0.13
Male >19	1.42	0.42	2.43	-0.18	-0.30	-0.06	0.003	1.08	0.08	2.08	0.03	0.04	0.00	0.07	0.02	0.001
Female>19	9.03	6.82	11.24	0.06	-0.20	0.32	0.67	0.58	-1.61	2.77	0.60	0.05	-0.02	0.12	0.13	0.99
Age <1	1.11	0.60	1.63	-0.07	-0.13	-0.01	0.02	-0.31	-0.82	0.20	0.23	-0.01	-0.02	0.01	0.48	0.04
Age 1-5	0.64	0.16	1.13	-0.07	-0.12	-0.01	0.02	-0.05	-0.53	0.43	0.85	0.001	-0.01	0.02	0.87	0.03
Age 6-19	3.49	2.49	4.50	0.06	-0.06	0.18	0.31	-0.14	-1.13	0.86	0.79	-0.01	-0.04	0.02	0.57	0.26
Age 20-45	8.92	6.73	11.11	0.01	-0.25	0.27	0.95	0.92	-1.26	3.09	0.41	0.06	-0.01	0.13	0.08	0.70
Age 46-65	0.82	0.06	1.57	-0.07	-0.16	0.02	0.13	0.50	-0.24	1.24	0.19	0.01	-0.01	0.04	0.28	0.08
Age >65	0.72	0.10	1.34	-0.07	-0.14	0.00	0.07	0.24	-0.37	0.85	0.44	0.02	0.00	0.04	0.09	0.03
Maternal and neonatal	9.74	7.58	11.89	0.27	0.01	0.52	0.04	0.23	-1.91	2.36	0.83	-0.03	-0.10	0.03	0.32	0.03
Non-communicable diseases	3.07	1.79	4.34	-0.13	-0.28	0.02	0.09	1.11	-0.15	2.38	0.08	-0.02	-0.06	0.02	0.24	0.19
Communicable diseases	5.38	3.78	6.97	-0.18	-0.37	0.01	0.06	0.54	-1.04	2.12	0.50	-0.02	-0.07	0.03	0.37	0.11
Injuries	1.83	1.13	2.54	-0.11	-0.19	-0.02	0.01	0.46	-0.24	1.16	0.20	-0.02	-0.04	0.001	0.06	0.06
Respiratory	0.14	-0.42	0.71	-0.09	-0.16	-0.03	0.01	0.23	-0.32	0.79	0.41	0.02	0.000	0.036	0.04	0.001

For peer review only

For peer review only - http://bmjopen.bmj.com/site/about/guidelines.xhtml

Supplementary Table 4: Risk of death among admissions: logistic regression models with and without adjustment for sex, age category and diagnosis.*

	Odds ratio	95% Confidence Interval		P value
**Model 1, not adjusted for case mix**				
Weekly change in risk of death pre-lockdown	1.03	0.97	1.09	0.37
Step change in risk of death at lockdown	0.49	0.29	0.82	0.01
Weekly change in risk of death post-lockdown	1.03	1.01	1.04	0.002*
**Model 2, adjusted for case mix**				
Weekly change in risk of death pre-lockdown	1.10	1.02	1.18	0.01
Step change in risk of death at lockdown	0.31	0.17	0.56	<0.001
Weekly change in risk of death post-lockdown	1.03	1.01	1.05	0.001*
Female	0.73	0.56	0.94	0.02
Age	1.02	1.02	1.03	<0.001
Communicable disease	2.33	1.46	3.71	<0.001
Neoplasm	3.32	1.52	7.26	0.003
Blood disorder	1.96	1.01	3.83	0.05
Endocrine	0.95	0.48	1.90	0.89
Mental health	0.41	0.13	1.35	0.14
Neurological	2.33	0.99	5.49	0.05
Otological	2.59	0.33	20.63	0.37
Circulatory	3.11	1.84	5.24	<0.001
Respiratory	2.47	1.49	4.08	<0.001
Gastroenterology	2.75	1.42	5.31	0.003
Dermatology	0.73	0.30	1.80	0.49
Musculoskeletal	0.49	0.07	3.62	0.49
Renal and urology	1.48	0.78	2.82	0.23
Breast and gynaecology	0.27	0.04	2.06	0.21
Maternal	1.00	0.00	0.00	<0.001
Neonatal	2.31	1.15	4.66	0.02
Injuries	0.31	0.09	1.04	0.06

* Lockdown-time interaction: P=0.325 for model 1 and P=0.126 for model 2

For peer review only

Supplementary Figure 1. LOWESS curves of daily admissions by age and sex, 2019-2020

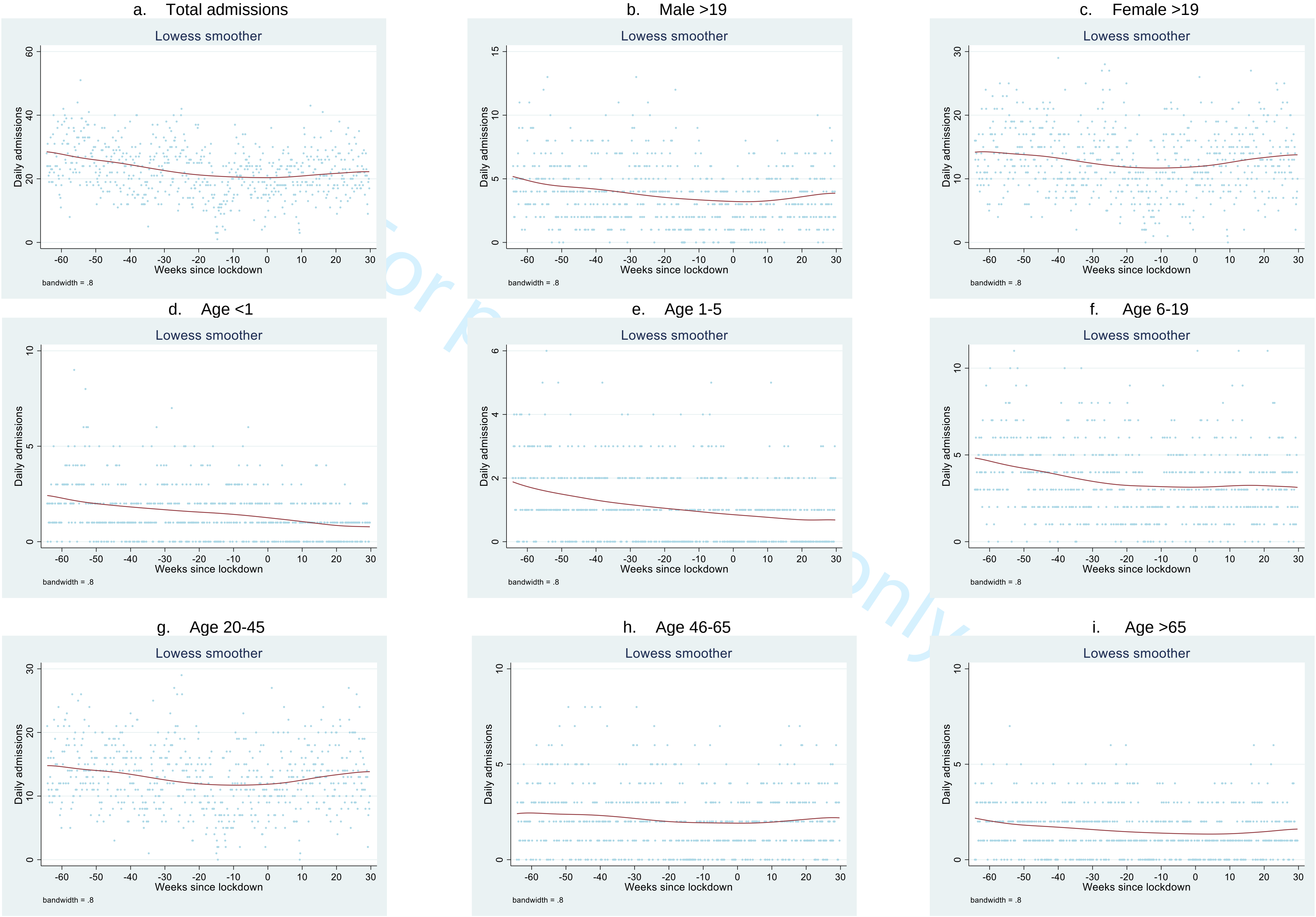

Supplementary Figure 2. LOWESS curves of daily admissions by diagnostic category, 2019-2020

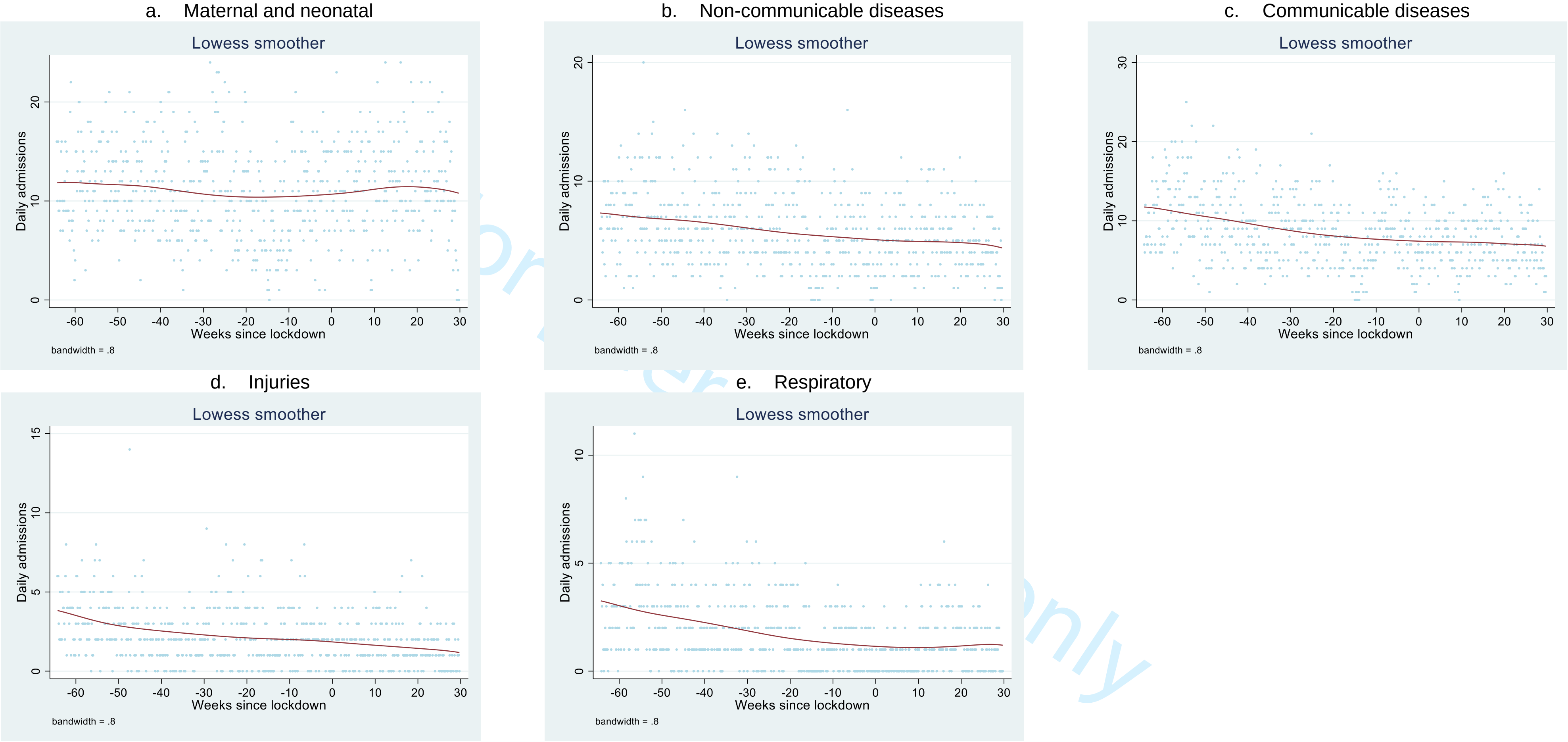

Supplementary Figure 3. LOWESS curve of probability of death by week of admission

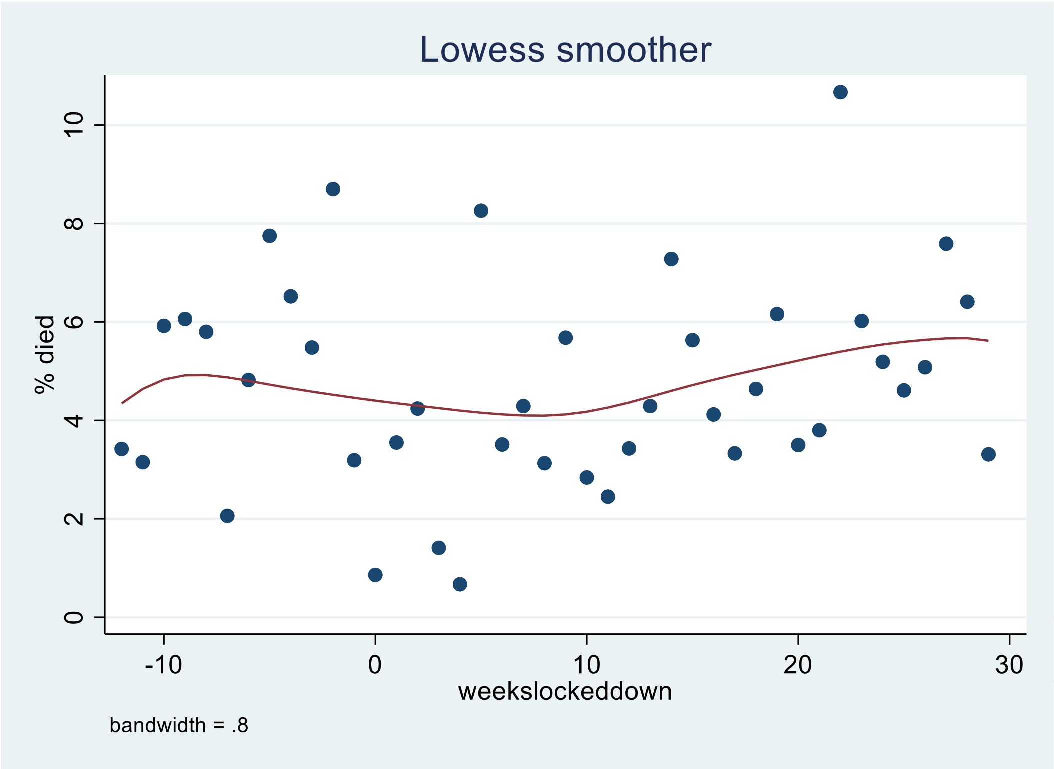

view only

STROBE 2007 (v4) Statement—Checklist of items that should be included in reports of *cohort studies*

Section/Topic	Item #	Recommendation	Reported on page #
**Title and abstract**	1	(*a*) Indicate the study's design with a commonly used term in the title or the abstract	1
		(*b*) Provide in the abstract an informative and balanced summary of what was done and what was found	2
**Introduction**			
Background/rationale	2	Explain the scientific background and rationale for the investigation being reported	4
Objectives	3	State specific objectives, including any prespecified hypotheses	5
**Methods**			
Study design	4	Present key elements of study design early in the paper	5-6
Setting	5	Describe the setting, locations, and relevant dates, including periods of recruitment, exposure, follow-up, and data collection	5-6
Participants	6	(*a*) Give the eligibility criteria, and the sources and methods of selection of participants. Describe methods of follow-up	5-6
		(*b*) For matched studies, give matching criteria and number of exposed and unexposed	
Variables	7	Clearly define all outcomes, exposures, predictors, potential confounders, and effect modifiers. Give diagnostic criteria, if applicable	5-6
Data sources/ measurement	8*	For each variable of interest, give sources of data and details of methods of assessment (measurement). Describe comparability of assessment methods if there is more than one group	*5-6*
Bias	9	Describe any efforts to address potential sources of bias	5-6
Study size	10	Explain how the study size was arrived at	5
Quantitative variables	11	Explain how quantitative variables were handled in the analyses. If applicable, describe which groupings were chosen and why	6-7
Statistical methods	12	(*a*) Describe all statistical methods, including those used to control for confounding	6-7
		(*b*) Describe any methods used to examine subgroups and interactions	6-7
		(*c*) Explain how missing data were addressed	8
		(*d*) If applicable, explain how loss to follow-up was addressed	n/a
		(*e*) Describe any sensitivity analyses	6-7
**Results**			

For peer review only

Participants	13*	(a) Report numbers of individuals at each stage of study—eg numbers potentially eligible, examined for eligibility, confirmed eligible, included in the study, completing follow-up, and analysed	7
		(b) Give reasons for non-participation at each stage	n/a
		(c) Consider use of a flow diagram	n/a
Descriptive data	14*	(a) Give characteristics of study participants (eg demographic, clinical, social) and information on exposures and potential confounders	5, 7-8
		(b) Indicate number of participants with missing data for each variable of interest	n/a
		(c) Summarise follow-up time (eg, average and total amount)	n/a
Outcome data	15*	Report numbers of outcome events or summary measures over time	7
Main results	16	(*a*) Give unadjusted estimates and, if applicable, confounder-adjusted estimates and their precision (eg, 95% confidence interval). Make clear which confounders were adjusted for and why they were included	8-11, supplementary tables
		(*b*) Report category boundaries when continuous variables were categorized	11
		(*c*) If relevant, consider translating estimates of relative risk into absolute risk for a meaningful time period	Supplementary tables
Other analyses	17	Report other analyses done—eg analyses of subgroups and interactions, and sensitivity analyses	8-11
**Discussion**			
Key results	18	Summarise key results with reference to study objectives	12
**Limitations**			
Interpretation	20	Give a cautious overall interpretation of results considering objectives, limitations, multiplicity of analyses, results from similar studies, and other relevant evidence	12-14
Generalisability	21	Discuss the generalisability (external validity) of the study results	14
**Other information**			
Funding	22	Give the source of funding and the role of the funders for the present study and, if applicable, for the original study on which the present article is based	18

*Give information separately for cases and controls in case-control studies and, if applicable, for exposed and unexposed groups in cohort and cross-sectional studies.

Note: An Explanation and Elaboration article discusses each checklist item and gives methodological background and published examples of transparent reporting. The STROBE checklist is best used in conjunction with this article (freely available on the Web sites of PLoS Medicine at http://www.plosmedicine.org/, Annals of Internal Medicine at http://www.annals.org/, and Epidemiology at http://www.epidem.com/). Information on the STROBE Initiative is available at www.strobe-statement.org.

For peer review only

BMJ Open

Effect of COVID-19 lockdown on hospital admissions and mortality in rural KwaZulu-Natal, South Africa: interrupted time series analysis

Journal:	*BMJ Open*
Manuscript ID	bmjopen-2020-047961.R1
Article Type:	Original research
Date Submitted by the Author:	30-Jan-2021
Complete List of Authors:	McIntosh, Amy; University of East Anglia Faculty of Medicine and Health Sciences,<br>Bachmann, Max; University of East Anglia Faculty of Medicine and Health Sciences<br>Siedner, Mark; Africa Health Research Institute; Harvard Medical School<br>Gareta, Dickman; Africa Health Research Institute<br>Seeley, Janet; Africa Health Research Institute; London School of Hygiene and Tropical Medicine, Global Health and Development<br>Herbst, Kobus; Africa Health Research Institute
**Primary Subject Heading**:	Public health
Secondary Subject Heading:	Global health, Epidemiology, Health services research, Paediatrics, Infectious diseases
Keywords:	PUBLIC HEALTH, COVID-19, PAEDIATRICS, EPIDEMIOLOGY

SCHOLARONE™
Manuscripts

I, the Submitting Author has the right to grant and does grant on behalf of all authors of the Work (as defined in the below author licence), an exclusive licence and/or a non-exclusive licence for contributions from authors who are: i) UK Crown employees; ii) where BMJ has agreed a CC-BY licence shall apply, and/or iii) in accordance with the terms applicable for US Federal Government officers or employees acting as part of their official duties; on a worldwide, perpetual, irrevocable, royalty-free basis to BMJ Publishing Group Ltd ("BMJ") its licensees and where the relevant Journal is co-owned by BMJ to the co-owners of the Journal, to publish the Work in this journal and any other BMJ products and to exploit all rights, as set out in our licence.

The Submitting Author accepts and understands that any supply made under these terms is made by BMJ to the Submitting Author unless you are acting as an employee on behalf of your employer or a postgraduate student of an affiliated institution which is paying any applicable article publishing charge ("APC") for Open Access articles. Where the Submitting Author wishes to make the Work available on an Open Access basis (and intends to pay the relevant APC), the terms of reuse of such Open Access shall be governed by a Creative Commons licence – details of these licences and which Creative Commons licence will apply to this Work are set out in our licence referred to above.

Other than as permitted in any relevant BMJ Author's Self Archiving Policies, I confirm this Work has not been accepted for publication elsewhere, is not being considered for publication elsewhere and does not duplicate material already published. I confirm all authors consent to publication of this Work and authorise the granting of this licence.

For peer review only

Effect of COVID-19 lockdown on hospital admissions and mortality in rural KwaZulu-Natal, South Africa: interrupted time series analysis

AUTHORS

Amy McIntosh

Norwich Medical School, Faculty of Medicine and Health Science, University of East Anglia, Norwich, UK

https://orcid.org/0000-0003-3690-2914

Max Bachmann

Norwich Medical School, Faculty of Medicine and Health Science, University of East Anglia, Norwich, UK

https://orcid.org/0000-0003-1770-3506

Mark J Siedner

Africa Health Research Institute, KwaZulu-Natal, South Africa

Department of Medicine, Harvard Medical School, Boston, Massachusetts, USA

http://orcid.org/0000-0003-3506-842X

Dickman Gareta

https://orcid.org/0000-0002-4004-5655

Africa Health Research Institute, Kwa-Zulu Natal, South Africa

Janet Seeley

Africa Health Research Institute, KwaZulu-Natal, South Africa

Department of Global Health and Development, London School of Hygiene and Tropical Medicine, London, UK

https://orcid.org/0000-0002-0583-5272

Kobus Herbst

Africa Health Research Institute, KwaZulu-Natal, South Africa

DSI-MRC South African Population Research Infrastructure Network, South Africa.

https://orcid.org/0000-0002-5436-9386

Corresponding Author: Dr Amy McIntosh, A.Mcintosh@doctors.org.uk

Word Count:3594

For peer review only - http://bmjopen.bmj.com/site/about/guidelines.xhtml

ABSTRACT

Objective To assess the effect of lockdown during the 2020 COVID-19 pandemic on daily all-cause admissions, and by age and diagnosis sub-groups, and odds of all-cause mortality in a hospital in rural KwaZulu-Natal (KZN).

Design Observational cohort.

Setting Referral hospital for 17 primary care clinics in uMkhanyakude district.

Participants Data collected by the Africa Health Research Institute on all admissions from 1st January to 20th October 2020: 5848 patients contributed to 6173 admissions.

Exposure Five levels of national lockdown in South Africa from 27th March 2020, with restrictions decreasing from levels 5 to 1 respectively.

Outcome Measures Changes and trends in daily all-cause admissions and risk of in-hospital mortality before, and at each stage of lockdown, estimated by Poisson and logistic interrupted time series regression, with stratification for age, sex, and diagnosis.

Results Daily admissions decreased during level 5 lockdown for infants (incidence rate ratio (IRR) compared to pre-lockdown 0.63, 95% confidence interval (CI) 0.44-0.90), 1-5 year-olds (IRR 0.43, 95% CI 028-0.65) and for respiratory diagnoses (IRR 0.57,95% CI 0.36-0.90). From level 4 to level 3, total admissions increased (IRR 1.17, 95% CI 1.06-1.28) as well as for men >19 years (IRR 1.50, 95% CI 1.17-1.92) and respiratory diagnoses (IRR 4.26, 95% CI 2.36-7.70). Among patients admitted to hospital, odds of death decreased during level 5 compared to pre-lockdown (adjusted odds ratio (aOR) 0.48; 95% CI 0.28-0.83), then increased in later stages.

Conclusions Level 5 lockdown is likely to have prevented the most vulnerable population, children under 5 years and those more severely ill, accessing hospital care in rural KZN, as reflected by the drop in admissions and odds of mortality. Subsequent increases in admissions, and in odds of death in hospital, could be due to improved and delayed access to hospital as restrictions were eased.

KEYWORDS

COVID-19, universal healthcare, secondary care, hospital admissions, healthcare utilisation, public health

For peer review only

STRENGTHS AND LIMITATIONS OF THIS STUDY

- Interrupted time series regression provides original insight into impact of COVID-19 lockdown on hospital admissions and mortality in South Africa
- This paper complements recently published evidence from the same catchment to give a fuller picture of access to multiple levels of health care in one population
- Hospital data provide only indirect evidence of access to hospital care and reasons for changes in access
- Using data from one hospital in a rural, low-middle income setting in South Africa limits generalisability to other settings
- Small sample limits statistical power to detect changes in sub-groups

For peer review only

BACKGROUND

The COVID-19 global pandemic has caused huge disruption to health systems worldwide. As numbers of cases of the novel coronavirus rose throughout 2020, health systems focussed resources onto COVID-19. This led to global reductions in resources for non-COVID-19 services [1–4]. Concurrently, governments attempted to reduce community transmission with lockdowns, restricting movement and activities of populations. Such restrictions have had far-reaching impacts on all aspects of society, including economies, education, unemployment, inequalities and health [5–7].

The effects of these measures on progress to reach universal health coverage globally are largely unknown, but it is likely that progress in low and middle income countries was adversely affected due to weak and under-resourced health systems serving populations with high burdens of both communicable (CDs) and non-communicable disease (NCDs), as well as social and economic deprivation [8]. Concerns have been voiced about impacts on HIV care, family planning services, and maternal and child health [9–11].

A potential consequence of lockdowns is reduced utilisation for non-COVID-19 conditions. Excess mortality during the pandemic that is not explained by direct mortality from COVID-19 supports this concern [12,13]. Predicted effects, both through reduced health service provision and reduced utilisation, include higher rates of mortality from vaccine-preventable disease, HIV, tuberculosis, in children aged under five years and pregnant women [14–17].

South Africa implemented a level 5 lockdown order at midnight 26th March 2020 [18]. This prohibited non-essential movement, and closure of non-essential businesses, schools, and services. Health workers were exempt from movement restrictions, as was accessing health services. However, this led to significant reductions in availability of transport, and reductions to income, particularly for informal or non-essential workers. Restrictions were eased incrementally over time (Figure 1) [19–21]. The lowest level of lockdown, level 1, was initiated on 21st September[22] and continued beyond the end of this study (20th October 2020).

Lockdown restrictions applied nationwide, including KwaZulu-Natal province, despite COVID-19 cases and deaths only increasing after the end of May[23] and peaking around the 25th July. COVID-19 deaths peaked around 8th August. By 20th October daily cases and deaths in the province had decreased to less than 10 and 1 per million population, respectively. The effects of levels 5, 4 and 3 lockdown on utilisation of primary care facilities in this mostly rural KwaZulu-Natal subdistrict have been reported by Siedner and colleagues[24]. They found that level 5 lockdown was associated with a significant reduction

For peer review only - http://bmjopen.bmj.com/site/about/guidelines.xhtml

in visits to primary care clinics for child health and in under 5 year-olds, but not for total visits or for adult care.

In this study we investigated the effects of the lockdown measures during the COVID-19 pandemic on admissions to, and deaths in, the referral hospital serving the same clinics and population as the latter study. We focussed particularly on admissions in sub-groups stratified by age, sex, and diagnosis, as indicators of access to hospital care. We also compared the risk of in-hospital mortality before and after lockdown as an indicator of the severity of presenting illnesses. We hypothesised that lockdown measures would lead to reduced hospital admissions, because of reduced mobility, and would increase as lockdown restrictions were eased. We also hypothesised that risk of in-hospital mortality would increase if people with less severe illness were selectively deterred from going to hospital or would decrease if those with more severe illness were selectively deterred.

METHODS

Study Population

We performed secondary analysis of data collected by the Africa Health Research Institute (AHRI), located in a sub-district of the northern KwaZulu-Natal, uMkhanyakude district in South Africa. It is a largely rural district, which is socio-economically deprived, falling in the bottom 10 of all districts in South Africa for education levels, employment, access to piped water and sanitation in the home[25]. The study population was patients admitted to Hlabisa hospital from 1st January 2020 to 20th October 2020. It is the main referral hospital for 17 clinics in the sub-district and is in an urban centre. Only 5% of the population hold medical insurance, so are mostly dependent on the hospital and clinics for their health care[25].

Data Collection

Data for each admission were captured prospectively by AHRI clinical research workers based in the hospital. These include demographic data for each patient, as well as admission, discharge, and death diagnoses, coded according to International Classification of Disease (ICD-10) by trained nurses, and recorded on an electronic database[26]. Women admitted for elective Caesarean-section, antenatal care, or other intrapartum care were included, excluding those admitted for normal vaginal deliveries.

Statistical Analysis

Total admissions for the study period were stratified by age, sex, and discharge diagnosis to demonstrate the typical case-mix at the hospital. To identify suitable regression models for

For peer review only - http://bmjopen.bmj.com/site/about/guidelines.xhtml

estimating effects of lockdown we first graphically plotted numbers of daily admissions and probability of death over time with LOWESS smoothing.

We used interrupted time series linear regression to estimate the effects of lockdown on numbers of daily admissions to hospital. We first calculated the number of admissions each day then analysed the aggregated data at day level. These analyses were carried out for all admissions and for subgroups defined by age, sex, and diagnosis. We used two sets of regression models to estimate the effect of lockdown on hospital utilisation, one estimating step changes in daily admissions or odds of death during each level of lockdown compared to pre-lockdown, and the other estimating linear time trends over the entire lockdown period.

To estimate absolute changes in mean numbers of daily admissions between each level of lockdown, compared to the pre-lockdown period, we used the following model:

$$Yt = \beta_0 + \beta_1 L_5 + \beta_2 L_4 + \beta_3 L_3 + \beta_4 L_2 + \beta_5 L_1 + \beta_{6-11} Z$$

Yt is the daily number of hospital admissions. β_0 is the mean daily number of visits or admissions immediately before lockdown (the regression intercept). L_5, L_4, L_3, L_2 *and* L_1 are binary dummy variables indicating each stage of lockdown, and their coefficients ($\beta_1 - \beta_5$) are mean differences from the pre-lockdown period; differences between successive levels were the differences between the corresponding coefficients. *Z* is a vector of dummy variables representing day of week.

The regression equation to estimate the continuous time trend before and after level 5 lockdown, and step change at the start of level 5, was as follows:

$$Yt = \beta_0 + \beta_1 T + \beta_2 X_t + \beta_3 TX_t + \beta_4 Z$$

Yt, β_0, and *Z* are the same as in the previous equation. β_1 is the weekly change in daily admissions before lockdown (that is, coefficient of the slope before lockdown) and T represents the number of weeks before or after lockdown. β_2 is the step change in number of admissions immediately after level 5 lockdown on 27th March 2020. X_t represents the pre- and post-lockdown periods coded as a binary variable. β_3 is the change in trend after lockdown. TX_t is the time-lockdown interaction term; $\beta_1 + \beta_3$ is thus the time trend after lockdown. We used the results of each analysis to graphically plot predicted continuous time trends and step changes.

We fused Poisson regression to estimate relative changes and then used linear regression to estimate absolute changes in daily admissions associated with lockdown for both models. We plotted frequency distributions of residuals of linear regression models, overall and in

For peer review only - http://bmjopen.bmj.com/site/about/guidelines.xhtml

subgroups, to assess their normality. Predicted values from the linear regression models with continuous time trends were plotted against number of weeks before and after lockdown.

Odds of in-hospital mortality among admitted patients was investigated using equivalent logistic regression models (with Yt representing log odds of death). These analyses were at the level of individual admission, with robust adjustment for intra-person correlation of outcomes during repeated admissions. Age, sex and diagnosis (ICD-10 chapter) were added as covariates instead of carrying out separate analyses for each subgroup. To graphically plot changes in probability of death before and after the start of level 5 lockdown we calculated the probability of death each week, using a linear regression model equivalent to the second equation above.

As a sensitivity analysis, to assess the effect of seasonal confounding we extended the study period to the start of 2019 and added calendar month as covariates to each model.

We use a 5% significance level and 95% confidence intervals. Analysis was carried out with Stata 16.0.

Ethical Issues

Ethical approval was granted for AHRI to collect and use data from patients attending the clinics and hospital by the University of KwaZulu Natal Biomedical Research Ethics Committee: number and title BE290/16 “A longitudinal population-based platform for epidemiology and intervention research”**.** This was a secondary analysis on this anonymised data set with permission from AHRI, so further ethical approval was not required.

Patient and Public Involvement

There was no patient or public involvement in this study.

RESULTS

There were 6173 admissions by 5848 individuals to the study hospital from 1st January 2020 to 20th October 2020 (Table 1). 4.7% (n=291) of admissions resulted in death. 59.4% of all admissions were in 20-45 year-olds (n=3664), and more admissions were in females compared to males (77.6% vs. 22.4%, n=4788 vs. n=1385). The most common reasons for admission were maternal or neonatal conditions (32.0% n=3271), communicable diseases (CDs) (21.6% n=2203), and non-communicable diseases (NCDs) (14.2% n=1455). Ninety-two admissions were identified as having COVID-19, of which 66.3% occurred during level 3 lockdown (n=61) and 21.7% (n=20) during level 2 lockdown. LOWESS plots of crude time

For peer review only - http://bmjopen.bmj.com/site/about/guidelines.xhtml

trends in admissions and mortality during 2019 and 2020 are shown graphically in Supplementary Figures 1-3.

Incidence rate ratios of daily admissions between each level of lockdown are shown in Table 2. All-cause daily admissions did not change significantly between levels, although they increased slightly from levels 4 to 3 (incidence rate ratio (IRR) 1.17, 95% confidence interval (CI) 1.06-1.28).

However, there were statistically significant changes among subgroups of patients. Daily admissions decreased significantly during level 5 lockdown for infants (IRR 0.63, 95% CI 0.44-0.90), 1-5 year-olds (IRR 0.43, 95% CI 028-0.65) and for respiratory diagnoses (IRR 0.57, 95% CI 0.36-0.90). In infants this represents a reduction in the incidence rate from 1.46 to 0.91 admissions per day, which did not recover by the end of the study, with 0.42 admissions per day during level 1 lockdown. The same trend was seen in 1-5 year olds, with incidence rate before lockdown 1.07 per day, reducing to 0.43 admissions per day during level 5, and not recovering by level 1, with incidence rate 0.61 per day. The most common reasons for admission in infants were neonatal conditions (42.6%), gastroenteritis (18.6%) and pneumonia (9.5%). In 1-5 year-olds they were injuries (32.1%), gastroenteritis (15.0%) and pneumonia (10.6%).

From level 4 to level 3, admissions increased significantly for men aged over 19 (IRR 1.50, 95% CI 1.17-1.92), 20-45 year-olds (IRR 1.19, 95% CI 1.05-1.34) and for respiratory diagnoses (IRR 4.26, 95% CI 2.36-7.70).

From level 2 to level 1, admissions decreased significantly for maternal and neonatal diagnoses (IRR 0.74, 95% CI 0.64-0.86), NCDs (IRR 0.76, 95% CI 0.60-0.96) and injuries (IRR 0.54, 95% CI 0.34-0.86). As a sensitivity analysis, admissions from the 1st of October onwards were excluded to test if this finding was due to data collection or upload delays in ICD-10 coding. The decrease seen in NCDs persisted but became non-significant (IRR 0.79, 95% CI 0.56-1.11). Maternal and neonatal admissions no longer decreased (IRR 1.00, 95% CI 0.83–1.22). Admissions for injuries continued to decrease (IRR 0.43, 95% CI 0.19-0.94). There were no statistically significant changes in these sub-groups during earlier stages of lockdown. Daily number of admissions in 6-19, 45-65, over 65 year olds, and for adult women and CDs did not change significantly at any stage of lockdown.

Linear regression models of these step changes produced similar results (Supplementary Table 1). Residuals of linear regression models were approximately normally distributed. Poisson and linear regression models of continuous time trends before and after the start of level 5 lockdown produced similar results, although some differences became non-significant (Supplementary Tables 2 and 3, Figures 2 and 3).

For peer review only - http://bmjopen.bmj.com/site/about/guidelines.xhtml

Among patients admitted to hospital, odds of death decreased substantially and significantly during level 5 lockdown compared to pre-lockdown (adjusted odds ratio (OR) 0.48; 95% CI 0.28-0.83), then increased from level 5 to level 4 (OR 1.94, 95% CI 1.01-3.71), did not change from level 4 to level 3 (OR 0.84, 95% CI 0.51-1.39), increased from level 3 to level 2 (OR 1.74, 95% CI 1.15-2.63), then did not change during level 1 (Table 3). These changes were not affected by adjustment for age, sex or diagnosis, except that adjustment for diagnosis increased the odds ratios for levels 4 versus 5 and levels 2 versus 3 and made them statistically significant (Table 3, Supplementary Table 4). Figure 4 illustrates the large decrease in probability of death immediately after the start of level 5 lockdown which then increases over time.

Sensitivity analysis with adjustment for calendar month, including admissions from the start of 2019, did not substantially alter the direction of changes in daily admissions or effect size seen during lockdown. For example, admissions in children age 1-5 still decreased significantly at level 5 lockdown (IRR 0.45, 95% CI 0.29 - 0.68) when data from 2019 was included in analysis. However, these results are not included as LOWESS plots identified an overall decrease in admissions since the start of 2019 and this may have exaggerated the effect size seen during lockdown.

For peer review only

For peer review only - http://bmjopen.bmj.com/site/about/guidelines.xhtml

Table 1. Numbers (%) of admissions to and deaths in Hlabisa hospital, rural KwaZulu Natal, South Africa, from January 2020-September 2020 according to sex, age and diagnosis.

	**Total** n (% of column total)	**Male** n (% of row total)	**Female** n (% of row total)	**Age <1** n (%of row total)	**Age 1-5** n (% of row total)	**Age 6-19** n (% of row total)	**Age 20-45** n (% of row total)	**Age 46-65** n (% of row total)	**Age>65** n (% of row total)
**Total** n (%)	6173 (100.0)*	1385 (22.4)	4788 (77.6)	334 (5.4)	242 (3.9)	951 (15.4)	3664 (59.4)	580 (9.4)	402(6.5)
**Maternal and neonatal** n (%)	3271 (32.0)	78 (2.4)	3193 (97.6)	145 (4.4)	7 (0.2)	662 (20.2)	2446 (74.8)	6 (0.2)	5 (0.2)
**Non-communicable diseases** n (%)	1455 (14.2)	568 (39.0)	887 (61.0)	59 (4.1)	70 (4.8)	109 (7.5)	517 (35.5)	386 (26.5)	314 (21.6)
**Communicable diseases** n (%)	2203 (21.6)	591 (26.8)	1612 (73.2)	129 (5.9)	103 (4.7)	110 (5.0)	1448 (65.7)	280 (12.7)	133 (6.04)
**Injuries** n (%)	496 (4.9)	302 (60.9)	194 (39.1)	19 (3.8)	83 (16.7)	104 (21.0)	225 (45.4)	46 (9.3)	19 (3.8)
**Respiratory** n (%)	323 (3.2)	151 (46.8)	172 (53.3)	42 (13.0)	34 (10.5)	15 (4.6)	92 (28.5)	69 (21.4)	71 (22.0)
**Other** n (%)	2786 (27.3)	556 (20.0)	2230 (80.0)	194 (7.0)	124 (4.5)	672 (35.5)	1531 (55.0)	158 (5.7)	107 (3.8)
**Deaths** n (%)	291 (4.7)	139 (47.8)	152 (52.2)	18 (6.2)	2 (0.7)	7 (2.4)	111 (38.14)	80 (27.5)	73 (25.1)

*Primary and secondary diagnosis used for each admission, therefore total of column % exceed 100%.

For peer review only

Table 2: Step changes in daily incidence of admissions at each level of lockdown: Poisson regression models adjusted for day of week

	Pre-lockdown admission daily incidence rate (95% CI)			IRR at level 5 vs pre-lockdown	95% CI		P value	IRR at level 4 vs 5	95% CI		P value	IRR at level 3 vs 4	95% CI		P value	IRR at level 2 vs 3	95% CI		P value	IRR at level 1 vs 2	95% CI		P value
Total	17.08	15.73	18.55	0.96	0.88	1.05	0.41	0.99	0.88	1.10	0.81	1.17*	1.06	1.28	0.001	1.02	0.94	1.11	0.67	1.02	0.94	1.11	0.67
Male >19	2.59	2.11	3.19	0.95	0.75	1.21	0.70	0.93	0.69	1.25	0.61	1.50*	1.17	1.92	0.001	0.98	0.80	1.21	0.87	0.93	0.72	1.21	0.59
Female>19	8.96	8.02	10.00	1.05	0.93	1.18	0.43	1.03	0.89	1.18	0.71	1.11	0.99	1.25	0.08	1.04	0.93	1.16	0.49	0.92	0.80	1.05	0.20
Age <1	1.46	1.04	2.03	0.63*	0.44	0.90	0.01	0.60	0.35	1.02	0.06	1.52	0.93	2.49	0.09	0.75	0.48	1.18	0.21	1.17	0.67	2.02	0.58
Age 1-5	1.07	0.99	1.17	0.43*	0.28	0.65	<0.001*	0.88	0.47	1.66	0.70	1.50	0.88	2.55	0.14	0.57*	0.33	0.98	0.04	1.75	0.94	3.28	0.08
Age 6-19	3.11	2.56	3.79	0.99	0.80	1.24	0.95	1.05	0.80	1.37	0.74	0.98	0.78	1.24	0.89	1.17	0.95	1.45	0.14	0.76	0.58	0.99	0.05
Age 20-45	9.13	8.18	10.18	1.08	0.96	1.21	0.20	0.95	0.83	1.10	0.50	1.19*	1.05	1.34	0.01	1.05	0.95	1.17	0.34	0.90	0.79	1.03	0.12
Age 46-65	1.29	0.96	1.72	0.89	0.65	1.21	0.44	1.24	0.86	1.78	0.26	1.22	0.91	1.64	0.18	0.77	0.58	1.03	0.08	1.16	0.82	1.65	0.40
Age >65	1.14	0.83	1.57	0.80	0.56	1.17	0.25	1.29	0.83	2.01	0.26	1.04	0.73	1.48	0.83	1.18	0.86	1.63	0.31	0.83	0.55	1.23	0.35
Maternal and neonatal	8.28	7.38	0.00	1.09	0.97	1.23	0.17	1.04	0.90	1.21	0.56	1.03	0.91	1.16	0.68	1.08	0.97	1.21	0.17	0.74	0.64	0.86	<0.001
NCDs	3.84	3.23	4.57	0.90	0.75	1.08	0.27	1.15	0.92	1.43	0.23	1.07	0.89	1.29	0.46	0.90	0.76	1.08	0.27	0.76	0.60	0.96	0.02
CDs	6.34	5.51	0.00	0.88	0.77	1.02	0.10	0.95	0.79	1.15	0.62	1.14	0.98	1.34	0.10	0.91	0.78	1.05	0.20	0.86	0.71	1.04	0.11
Injuries	2.56	2.01	3.00	0.80	0.59	1.08	0.14	0.83	0.56	1.24	0.37	1.34	0.95	1.88	0.10	0.88	0.64	1.20	0.42	0.54	0.34	0.86	0.01
Respiratory	0.70	0.47	1.06	0.57	0.36	0.90	0.02	0.59	0.30	1.20	0.15	4.26*	2.36	7.70	<0.001	0.58*	0.40	0.85	0.01	0.89	0.53	1.48	0.65

IRR incidence rate ratio
* statistically significantly different from pre-lockdown

Table 3: Step changes in odds of death at Hlabisa hospital at each level of lockdown: logistic regression models*

	Pre-lockdown odds (95% CI)			OR level 5 vs pre	95% CI		P value	OR level 4 vs 5	95% CI		P value	OR level 3 vs 4	95% CI		P value	OR level 2 vs 3	95% CI		P value	OR level 1 vs 2	95% CI		P value
Unadjusted	0.05	0.04	0.07	0.48	0.28	0.80	<0.001	1.81	0.97	3.38	0.06	0.98	0.62	1.54	0.92	1.40	0.96	2.04	0.08	0.98	0.62	1.53	0.92
Adjusted for day of week, Sex and Age	0.02	0.02	0.04	0.47	0.28	0.80	<0.001	1.75	0.93	3.28	0.08	0.96	0.59	1.54	0.85	1.42	0.95	2.12	0.09	0.97	0.60	1.57	0.91
Adjusted for day of week, sex, age and diagnosis	0.04	0.03	0.07	0.48	0.28	0.83	0.01	1.94	1.01	3.71	0.05	0.84	0.51	1.39	0.51	1.74	1.15	2.63	0.01	1.00	0.59	1.71	0.99

OR Odds ratio

* analysis at level of admission, with robust adjustment for intra-person correlation of outcome

For peer review only

DISCUSSION

In this analysis of hospital admission and hospital mortality in a mostly rural South African setting, national lockdown was not associated with reductions in all-cause daily admissions. Total admissions, and of adult men, increased from level 4 to level 3. Numbers of admissions in adult women, over 45 year olds, 6-19 year olds, and for communicable diseases, were mostly unchanged after lockdown. However, admissions for children under five fell sharply during level 5 lockdown and remained low. During the final level 1 lockdown, admissions for maternal and neonatal conditions, non-communicable diseases and injuries dropped significantly compared to level 2, despite no significant changes during earlier levels of lockdown. Risk of in-hospital mortality halved during level 5 lockdown then increased to pre-lockdown rates.

These observed changes could be due to changes in the health of the local population, or to lockdown restrictions, or both. However, the magnitude of changes over a relatively short period and the small number of reported COVID-19 admissions, suggest that changes in access to hospital care are likely to have been influential. Reduced access to hospital during lockdown could be due to a combination of factors including mandatory restrictions on movement, fear of being infected, and inability to pay for transport due to lost income (hospital care is free to users). Availability of ambulance transport from home to hospital is limited. Expense of transport and travel time are known barriers to care in this population, and are likely to have been exacerbated by restrictions on public transport during lockdown. [27,28].

Our findings that all-cause hospital admissions did not fall, but that admissions of under five year olds decreased, were consistent with findings of Siedner and colleagues on primary care visitations in the same population[24]. However, in the present study under-five hospital admissions did not increase to normal after level 5, unlike primary care visits which did[24]. Reasons for reduced admissions in under-fives could include reduction in circulating viral illnesses following school closures, or reduction in injuries as children spend more time at home during the strict lockdown measures of level 5. It is unlikely that other seasonal factors such as respiratory viruses or reduction in Vitamin D production is the reason for the decline in admissions as lockdown started in the late summer months [29–31]. More concerningly, it could signify that unwell children were not being taken to hospital by caregivers for acute illnesses such as pneumonia or gastroenteritis, among the most common diagnoses in these age groups. This could explain the concurrent drop in respiratory admissions – particularly given that a large proportion of respiratory admissions were in children and most were due to pneumonia (Table 1). Reduced access to hospital during level 5 lockdown thus appears to

For peer review only - http://bmjopen.bmj.com/site/about/guidelines.xhtml

affect children more than other sub-groups, highlighting the vulnerability of children and their dependence on carers, who might have been concerned to protect their young children and themselves from COVID-19 infection.

Similar reductions in paediatric admissions for respiratory disease were reported during the early pandemic in China , and decreased emergency department attendance was reported in Victoria, Australia[32,33]. Modelling by Roberton and colleagues predicted that COVID-19 could cause at least 253,500 additional child deaths in low and middle income countries, 41% of which would be attributable to reduced acute care for childhood illnesses, such as antibiotic treatment of pneumonia[17]. The leading causes of child mortality in this low-income population are acute respiratory infections, HIV-related illness, neonatal pneumonia, diarrhoeal disease and pulmonary TB: also amongst the most common reasons for admission to Hlabisa hospital in under fives.[34] Reduction in access for treatable illnesses in children may thus have led to preventable mortality during lockdown.

These results suggest that admissions for over 45 years olds, communicable diseases and injuries remained stable despite the significant restrictions during lockdown. This is unlike other regions in South Africa, which saw reductions in surgical admissions in North West Province, and prenatal anti-retroviral pre-exposure prophylaxis visits in the Western Cape[35,36].

The KwaZulu-Natal provincial health department reported that by 11th April the cumulative incidence of COVID-19 in uMkhanyakude district was 21, with no deaths[37]. By 5th of August the cumulative incidence had risen to 1513 cases, accounting for 1.7% of cases in the province. The daily incidence rate was less than 50 cases per 100,000 population[38]. This was despite a peak in incidence of cases nationally in July[23]. Respiratory admissions in this study population (which excluded COVID-19) peaked during level 3, which coincides with the peak in COVID-19 incidence in KwaZulu-Natal. It is possible that undiagnosed COVID-19 presenting as pneumonia could account for some of this increase. The majority of admissions with a primary diagnosis of COVID-19 also occurred during level 3 at Hlabisa, and the combination of both diagnosed and undiagnosed COVID-19 may also partly account for the increase in admissions in men and 20-45 year olds during level 3 lockdown.

A possible explanation for the increase during level 3 could be migration into the area, if urban workers moved back to their rural homes when transport restrictions eased during level 3 lockdown[39]. However, Siedner and colleagues interrogated local longitudinal primary care attendance data and did not find evidence of substantial migration[24]. The temporary lifting of the ban on alcohol sales at the beginning of level 2 lockdown[20] could have led to an increase in alcohol-related admissions in these sub-groups, but admission for

For peer review only - http://bmjopen.bmj.com/site/about/guidelines.xhtml

injuries or NCDs, which are often alcohol-related, did not change significantly during level 2 lockdown. Admissions for NCDs and injuries also did not decrease significantly at the beginning of the lockdown when the alcohol ban commenced, as seen in the Western Cape and Cape Town [36,40].

A major finding of this study is the large reduction in all-cause mortality during level 5 lockdown, despite resilience in admissions numbers overall. This supports our hypothesis that the sickest people may have been disproportionately unable to access hospital care as needed and died at home as opposed to hospital, or presented late to hospital resulting in increasing mortality from level 4 onwards. Reductions in numbers of admissions in the sickest may have been offset by the observed increases in admissions of younger, healthier adults. The South African Medical Research Council reported 7729 excess deaths in KwaZulu-Natal from 6th May to 10th November 2020, compared to the same dates during previous years, which was much more than the number of COVID-19 cases reported[41]. These excess deaths were probably due both to undiagnosed COVID-19 and to undertreatment of other life-threatening conditions[41,42]. It is thus likely that reduced utilisation by the sickest may have resulted in preventable death outside of hospital – most concerningly this may be in the under fives, as this group was not accessing care for diseases which are a leading cause of mortality in the region. Further analysis of local demographic data on rates and place of death during lockdown may elucidate this finding.

Limitations of this study include only analysing admissions up till 20th October 2020, before the pandemic had resolved globally and the second waves of COVID-19 cases were beginning[43]. Therefore, the impact of the epidemic on long-term healthcare utilisation or hospital mortality cannot yet be assessed in this study. Our analysis is restricted by a relatively small data set from one district hospital, which may have limited the power of our study to detect effects of lockdown, particularly in sub-group analysis such as young children and the generalisability to other secondary care settings. While there were no other known significant events during lockdown in the region likely to have influenced utilisation of hospital care, we cannot exclude changes in disease epidemiology and subsequent healthcare demand, weather, or socio-cultural factors, which may account for the results.

Further work should focus on the impact of COVID-19 on access to children's health services. They appear to have a lower risk of acquiring and suffering from severe COVID-19[44–46], but the implications of strict lockdowns on young children's access and utilisation to healthcare in low income settings for acute illnesses is concerning. This study cannot evaluate the reasons for reduced healthcare utilisation in children in this population. However potential solutions may include pay-back schemes for emergency transport costs

For peer review only - http://bmjopen.bmj.com/site/about/guidelines.xhtml

(to offset the heightened costs during lockdown). The use of social media may also offer effective means of encouraging parents to seek care at local primary clinics, from where they could be transported to hospital if necessary.

REFERENCES

1 Kinross P, Suetens C, Gomes Dias J, *et al.* Rapidly increasing cumulative incidence of coronavirus disease (COVID-19) in the European Union/European Economic Area and the United Kingdom, 1 January to 15 March 2020. *Eurosurveillance* 2020;**25**:2000285. doi:10.2807/1560-7917.ES.2020.25.11.2000285

2 Italy's Health Care System Groans Under Coronavirus — a Warning to the World - The New York Times. https://www.nytimes.com/2020/03/12/world/europe/12italy-coronavirus-health-care.html (accessed 10 Nov 2020).

3 Dunn P, Allen L, Cameron G, *et al.* COVID-19 policy tracker | The Health Foundation. 2020.https://www.health.org.uk/news-and-comment/charts-and-infographics/covid-19-policy-tracker (accessed 10 Nov 2020).

4 World Health Organization. Statement on the third meeting of the International Health Regulations (2005) Emergency Committee regarding the outbreak of coronavirus disease (COVID-19). 1 May 2020. 2020. https://www.who.int/news/item/01-05-2020-statement-on-the-third-meeting-of-the-international-health-regulations-(2005)-emergency-committee-regarding-the-outbreak-of-coronavirus-disease-(covid-19) (accessed 28 Oct 2020).

5 Haffajee F. Post-Covid economy resembles a post-war landscape as joblessness climbs to highest ever. 2020.https://www.dailymaverick.co.za/article/2020-09-30-post-covid-economy-resembles-a-post-war-landscape-as-joblessness-climbs-to-highest-ever/ (accessed 10 Nov 2020).

6 Ewing J. Europe Risks a New Economic Downturn as Lockdowns Return. https://www.nytimes.com/2020/10/30/business/european-economy.html (accessed 10 Nov 2020).

7 United Nations Children's Fund. Education on hold | UNICEF Latin America and Caribbean. 2020. https://www.unicef.org/lac/en/reports/education-on-hold (accessed 10 Nov 2020).

8 Bulled N, Singer M. In the shadow of HIV & TB: A commentary on the COVID epidemic in South Africa. *Glob Public Health* 2020;**15**:1231–43. doi:10.1080/17441692.2020.1775275

9 Jiang H, Zhou Y, Tang W. Maintaining HIV care during the COVID-19 pandemic. *Lancet HIV* 2020;**7**:e308–9. doi:https://doi.org/10.1016/S2352-3018(20)30105-3

10 United Nations Family Planning Association. Interim Technical Note Impact of the COVID-19 Pandemic on Family Planning and Ending Gender-based Violence, Female Genital Mutilation and Child Marriage.

11 World Health Organization, United Nations Children's Fund. Protect the Progress: Rise, Refocus, Recover. 2020 Progress Report on the Every Women Every Child Global Strategy for Women's, Children's and Adolescents' Health (2016-2030). 2020. https://protect.everywomaneverychild.org/assets/img/2020-Progress-Report-on-the-EWEC-Global-Strategy-Final.pdf (accessed 29 Sep 2020).

For peer review only - http://bmjopen.bmj.com/site/about/guidelines.xhtml

12 Nogueira PJ, De Araújo Nobre M, Nicola PJ, *et al.* Excess Mortality Estimation During the COVID-19 Pandemic: Preliminary Data from Portugal. *Acta Med Port* 2020;**33**:376. doi:10.20344/amp.13928

13 Kontis V, Bennett JE, Rashid T, *et al.* Magnitude, demographics and dynamics of the effect of the first wave of the COVID-19 pandemic on all-cause mortality in 21 industrialized countries. *Nat Med* Published Online First: 2020. doi:10.1038/s41591-020-1112-0

14 Abbas K, Procter SR, van Zandvoort K, *et al.* Routine childhood immunisation during the COVID-19 pandemic in Africa: a benefit-risk analysis of health benefits versus excess risk of SARS-CoV-2 infection. *Lancet Glob Heal* 2020;**0**. doi:10.1016/S2214-109X(20)30308-9

15 Hogan AB, Jewell BL, Sherrard-Smith E, *et al.* Potential impact of the COVID-19 pandemic on HIV, tuberculosis, and malaria in low-income and middle-income countries: a modelling study. *Lancet Glob Heal* 2020;**0**. doi:10.1016/S2214-109X(20)30288-6

16 Jewell BL, Mudimu E, Stover J, *et al.* Potential effects of disruption to HIV programmes in sub-Saharan Africa caused by COVID-19: results from multiple mathematical models. *Lancet HIV* 2020;**7**:e629–40. doi:10.1016/S2352-3018(20)30211-3

17 Roberton T, Carter ED, Chou VB, *et al.* Early estimates of the indirect effects of the COVID-19 pandemic on maternal and child mortality in low-income and middle-income countries: a modelling study. *Lancet Glob Heal* 2020;**8**:e901–8. doi:10.1016/S2214-109X(20)30229-1

18 South Africa Department of Health. Disaster Management Act 2002 (Act No.57 of 2002) Alert level 5 during Coronavirus COVID-19 lockdown. Government Gazette No 43148:398. 25 March 2020.

19 South Africa Department of Health. Disaster Management Act 2002 (Act No.57 of 2002) Alert level 4 during Coronavirus COVID-19 lockdown. Government Gazette No 43258:480. 29 April 2020.

20 South Africa Department of Health. Disaster Management Act 2002 (Act No.57 of 2002) Alert level 3 during Coronavirus COVID-19 lockdown. Government Gazette No 43364:608. 28 May 2020.

21 South Africa Department of Health. Disaster Management Act 2002 (Act No.57 of 2002) Alert level 2 during Coronavirus COVID-19 lockdown. Government Gazette No 43620:891. 17 August 2020.

22 South Africa Department of Health. Disaster Management Act 2002 (Act No.57 of 2002) Alert level 1 during Coronavirus COVID-19 lockdown. Government Gazette No 43725:999. 18 September 2020.

23 National Institute for Communicable Diseases. COVID-19 WEEKLY EPIDEMIOLOGY BRIEF South Africa Week 42 2020. 2020.

24 Siedner MJ, Kraemer JD, Meyer MJ, *et al.* Access to primary healthcare during lockdown measures for COVID-19 in rural South Africa: an interrupted time series analysis. *BMJ Open* 2020;**10**. doi:10.1136/bmjopen-2020-043763

25 Massyn N, Day C, Ndlovu N, *et al.* District Health Barometer: District Health Profiles 2018/19. 2020.

26 World Health Organization. ICD-10 : international statistical classification of diseases

For peer review only - http://bmjopen.bmj.com/site/about/guidelines.xhtml

and related health problems : tenth revision, 2nd ed. 2004.https://icd.who.int/browse10/2019/en (accessed 25 Oct 2020).

27 Chimbindi N, Bor J, Newell M-L, *et al.* Time and Money: The True Costs of Health Care Utilization for Patients Receiving 'Free' HIV/Tuberculosis Care and Treatment in Rural KwaZulu-Natal. *J Acquir Immune Defic Syndr* 2015;**70**:e52–60. doi:10.1097/QAI.0000000000000728

28 Chimbindi N, Bärnighausen T, Newell ML. An integrated approach to improving the availability and utilisation of tuberculosis healthcare in rural South Africa. *South African Med J* 2013;**103**:237–40. doi:10.7196/SAMJ.6423

29 Olsen SJ, Azziz-Baumgartner E, Budd AP, *et al.* Decreased influenza activity during the COVID-19 pandemic-United States, Australia, Chile, and South Africa, 2020. *Am J Transplant* 2020;**20**:3681–5. doi:10.1111/ajt.16381

30 Li Y, Reeves RM, Wang X, *et al.* Global patterns in monthly activity of influenza virus, respiratory syncytial virus, parainfluenza virus, and metapneumovirus: a systematic analysis. *Lancet Glob Heal* 2019;**7**:e1031–45. doi:10.1016/S2214-109X(19)30264-5

31 Engelsen O. The relationship between ultraviolet radiation exposure and vitamin D status. *Nutrients* 2010;**2**:482–95. doi:10.3390/nu2050482

32 Cheek JA, Craig SS, West A, *et al.* Emergency department utilisation by vulnerable paediatric populations during the COVID-19 pandemic. *Emerg Med Australas* 2020;**32**:870–1. doi:10.1111/1742-6723.13598

33 Li H, Yu G, Duan H, *et al.* Changes in Children's Healthcare Visits During Coronavirus Disease-2019 Pandemic in Hangzhou, China. *J Pediatr* 2020;**224**:146. doi:10.1016/J.JPEDS.2020.05.013

34 Tlou B, Sartorius B, Tanser F. Space-time variations in child mortality in a rural South African population with high HIV prevalence (2000–2014). *PLoS One* 2017;**12**:e0182478.https://doi.org/10.1371/journal.pone.0182478

35 Davey DLJ, Bekker L-G, Mashele N, *et al.* PrEP retention and prescriptions for pregnant women during COVID-19 lockdown in South Africa. *Lancet HIV* Published Online First: August 2020. doi:10.1016/S2352-3018(20)30226-5

36 Moustakis J, Piperidis AA, Ogunrombi AB. The effect of COVID-19 on essential surgical admissions in South Africa: A retrospective observational analysis of admissions before and during lockdown at a tertiary healthcare complex. *South African Med J* 2020;**110**:910. doi:10.7196/SAMJ.2020.v110i9.15025

37 KwaZulu-Natal Department of Health, National Institute for Communicable Diseases. KwaZulu-Natal Department of Health COVID-19 Situation report :12 April 2020. 2020.

38 KwaZulu-Natal Department of Health, National Institute for Communicable Diseases. KwaZulu-Natal Department of Health COVID-19 Situational report: 5 August 2020. 2020.

39 Bank L. Covid-19 reveals migration links in South Africa's human economy. 2020.https://www.dailymaverick.co.za/article/2020-05-17-covid-19-reveals-migration-links-in-south-africas-human-economy/ (accessed 27 Oct 2020).

40 Reuter H, Jenkins LS, De Jong M, *et al.* Prohibiting alcohol sales during the coronavirus disease 2019 pandemic has positive effects on health services in South Africa. *African J Prim Heal care Fam Med* 2020;**12**:e1–4. doi:10.4102/phcfm.v12i1.2528

For peer review only - http://bmjopen.bmj.com/site/about/guidelines.xhtml

41 South African Medical Research Council. Report on Weekly Deaths in South Africa. 2020.https://www.samrc.ac.za/reports/report-weekly-deaths-south-africa (accessed 19 Oct 2020).

42 Dyer O. Covid-19: Excess deaths point to hidden toll in South Africa as cases surge. *BMJ* 2020;**370**:m3038. doi:https://dx.doi.org/10.1136/bmj.m3038

43 Looi M-K. Covid-19: Is a second wave hitting Europe? *BMJ* 2020;**371**:m4113. doi:10.1136/bmj.m4113

44 Viner RM, Mytton OT, Bonell C, *et al.* Susceptibility to SARS-CoV-2 Infection Among Children and Adolescents Compared With Adults: A Systematic Review and Meta-analysis. *JAMA Pediatr* Published Online First: 25 September 2020. doi:10.1001/jamapediatrics.2020.4573

45 Christophers B, Gallo Marin B, Oliva R, *et al.* Trends in clinical presentation of children with COVID-19: a systematic review of individual participant data. *Pediatr Res* Published Online First: 2020. doi:10.1038/s41390-020-01161-3

46 Ludvigsson JF. Systematic review of COVID-19 in children shows milder cases and a better prognosis than adults. *Acta Paediatr* 2020;**109**:1088–95. doi:10.1111/apa.15270

For peer review only

For peer review only - http://bmjopen.bmj.com/site/about/guidelines.xhtml

FUNDING STATEMENT

The Africa Health Research Institute is funded by Wellcome Trust award 201433/Z/16/Z.

COMPETING INTERESTS

None declared.

AUTHOR CONTRIBUTIONS

MB, MJS, JS and KH conceived the study aims and hypothesis.

KH and DG provided AHRI Hospital Information System data.

AM and MB designed the study and conducted the statistical analysis, with advice from MJS.

AM drafted the manuscript and prepared the tables, figures and final version for publication with substantial amendment and oversight from MB.

All authors discussed the results, critically revised the study and approved the final version of the manuscript.

ACKNOWLEDGMENTS

We thank the research assistants, nurses and staff at Hlabisa hospital and AHRI who contributed towards data collection. Thanks to Anita Edwards for proof-reading and support in preparing the manuscript for publication.

DATA SHARING

Statistical coding available on reasonable request from corresponding author. AHRI Hospital Information System data are available on request here: https://data.ahri.org/index.php/home.

For peer review only

LEGENDS

Table 1. Numbers (%) of admissions to and deaths in Hlabisa hospital, rural KwaZulu Natal, South Africa, from January 2020-September 2020 according to sex, age and diagnosis.

*Primary and secondary diagnosis used for each admission, therefore total of column % exceed 100%.

Table 2: Step changes in daily incidence of admissions at each level of lockdown: Poisson regression models adjusted for day of week

IRR: incidence rate ratio

* statistically significantly different from pre-lockdown

Table 3: Step changes in odds of death at Hlabisa hospital at each level of lockdown: logistic regression models*

* analysis at level of admission, with robust adjustment for intra-person correlation of outcome

OR: odds ratio

Figure 1: Levels of national covid-19 lockdown during 2020 in South Africa

Figure 2: Admissions to Hlabisa hospital from January 2020 to October 2020 for all reasons, by Sex and Age. Scatter plot represents number of admissions per day. Black line represents fitted linear regression model with adjustment for day of week, before and after lockdown. Dashed lines represent confidence intervals of the fitted model. Red line represents the beginning of lockdown level 5.

Figure 3: Admissions to Hlabisa hospital from January 2020 to October 2020, by discharge diagnosis. Scatter plot represents number of admissions per day. Black line represents fitted linear regression model with adjustment for day of week, before and after lockdown. Dashed lines represent confidence intervals of the fitted model. Red line represents the beginning of lockdown level 5.

Figure 4: Probability of death among all admissions each week: observed and predicted by interrupted times series linear regression model. Scatter plot represents probability of death each week. Black line represents fitted linear regression model with adjustment for day of week, before and after lockdown. Dashed lines represent confidence intervals of the fitted model. Red line represents the beginning of lockdown level 5.

For peer review only - http://bmjopen.bmj.com/site/about/guidelines.xhtml

Figure 1: Levels of national covid-19 lockdown during 2020 in South Africa				
**Level 5**	**Level 4**	**Level 3**	**Level 2**	**Level 1**
26th March – 31st April 2020	1st – 31st May 2020	1st June – 17th August 2020	18th August – 20th September 2020	21st September 2020 onwards
• Essential travel only<br>• Businesses closed<br>• Schools closed<br>• Transport restrictions<br>• Alcohol ban	• Some workplaces reopen<br>• Outdoor exercise permitted	• All workplaces & schools reopen<br>• Public transport restarts<br>• Physical distancing<br>• Alcohol ban lifted temporarily until 12th July	• Small gatherings of <50 people<br>• Leisure and social activities permitted<br>• Alcohol ban lifted	• Minimal restrictions<br>• All activities to be physically distanced and mask-wearing

For peer review only

Figure 2: Admissions to Hlabisa hospital from January 2020 to October 2020 for all reasons, by Sex and Age. Scatter plot represents number of admissions per day. Black line represents fitted linear regression model with adjustment for day of week, before and after lockdown. Dashed lines represent confidence intervals of the fitted model. Red line represents the beginning of lockdown level 5.

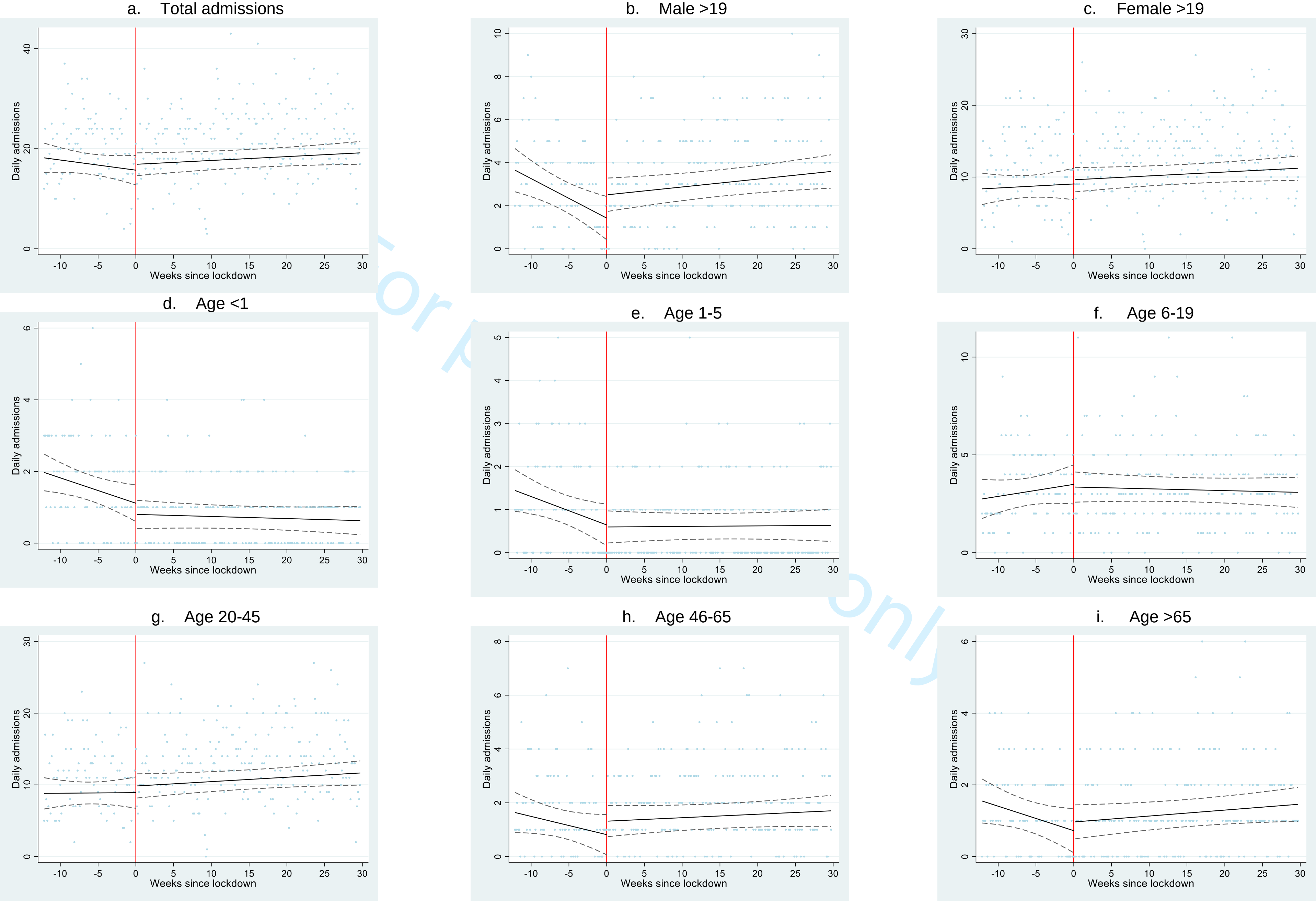

For peer review only

Figure 3: Admissions to Hlabisa hospital from January 2020 to October 2020, by discharge diagnosis. Scatter plot represents number of admissions per day. Black line represents fitted linear regression model with adjustment for day of week, before and after lockdown. Dashed lines represent confidence intervals of the fitted model. Red line represents the beginning of lockdown level 5.

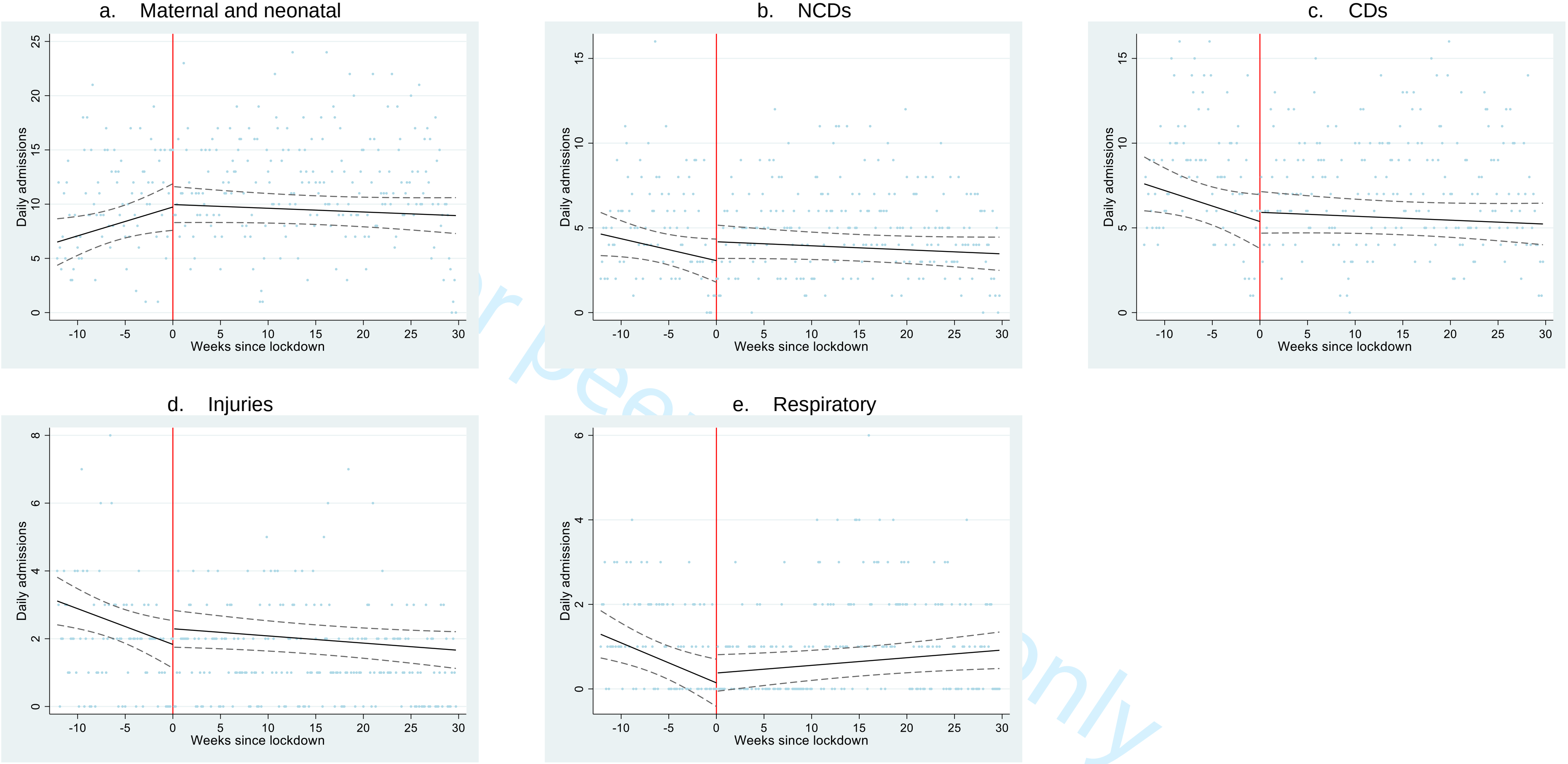

Figure 4: Probability of death among all admissions each week: observed and predicted by interrupted times series linear regression model. Black line represents fitted linear regression model with adjustment for day of week, before and after lockdown. Dashed lines represent confidence intervals of the fitted model. Red line represents the beginning of lockdown level 5.

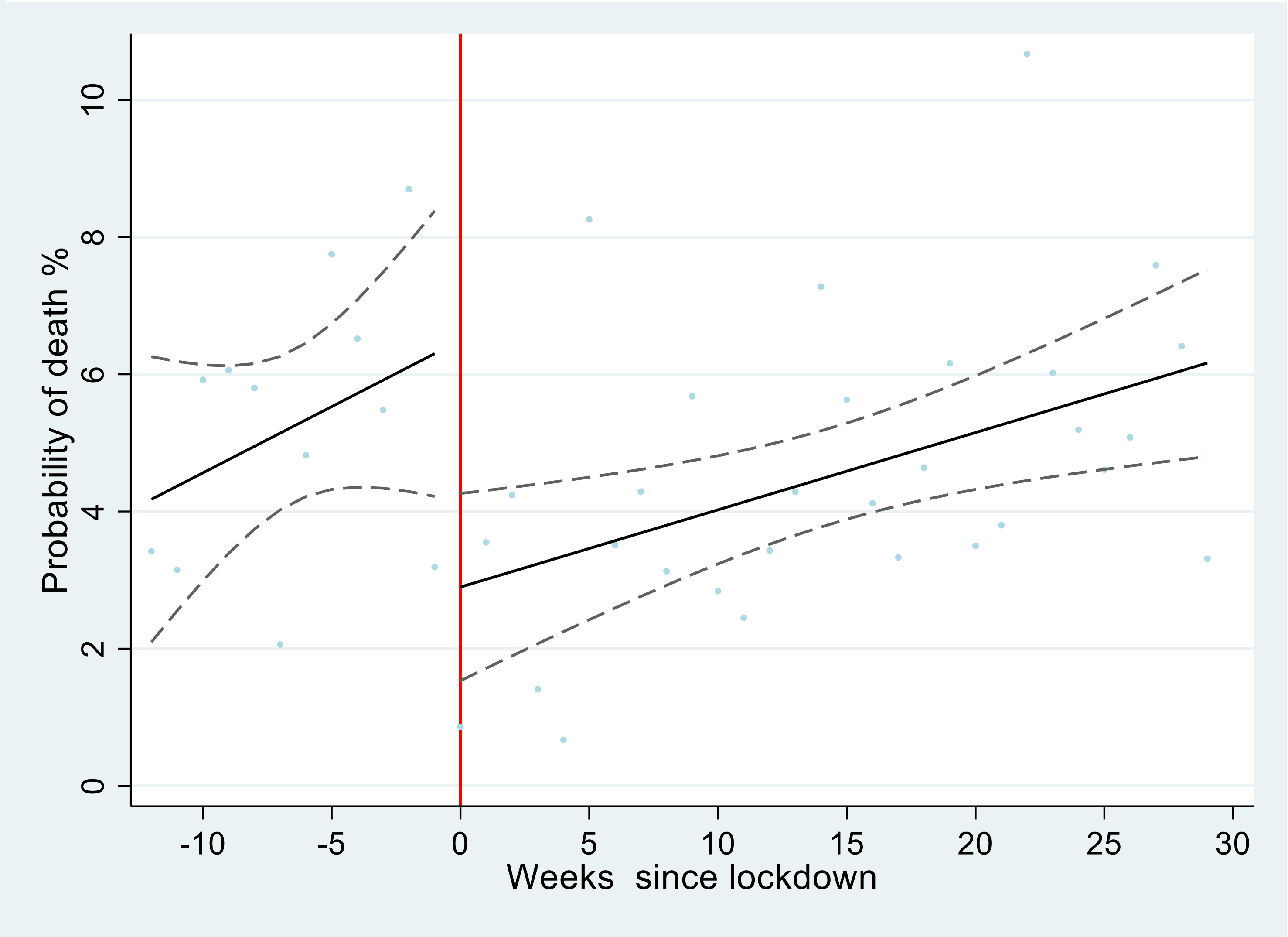

Supplementary Table 1: Changes in mean daily admissions to Hlabisa hospital at each stage of lockdown: linear regression models adjusted for day of week

	Pre-lockdown average daily admissions (95% CI)			Change at level 5	95% CI		P value	Change at level 4	95% CI		P value	Change at level 3	95% CI		P value	Change at level 2	95% CI		P value	Change at level 1	95% CI		P value
All admissions	16.96	14.93	19.00	-0.74	-3.02	1.53	0.52	-0.23	-3.03	2.57	0.87	3.18	0.77	5.59	0.01	0.40	-1.94	2.73	0.74	-1.88	-4.72	0.97	0.20
Male >19	2.55	1.85	3.26	-0.13	-0.92	0.66	0.74	-0.20	-1.17	0.77	0.68	1.27	0.44	2.11	<0.001	-0.07	-0.88	0.74	0.87	-0.25	-1.24	0.73	0.61
Female >19	8.70	7.17	10.23	0.54	-1.17	2.26	0.53	0.33	-1.78	2.44	0.76	1.36	-0.46	3.17	0.14	0.51	-1.25	2.27	0.57	-1.17	-3.31	0.97	0.28
Age <1	1.55	1.19	1.91	-0.64	-1.04	-0.23	<0.001	-0.44	-0.93	0.06	0.08	0.34	-0.09	0.76	0.12	-0.25	-0.66	0.17	0.24	0.12	-0.38	0.62	0.64
Age 1-5	1.05	0.71	1.39	-0.50	-0.88	-0.12	0.01	-0.07	-0.53	0.40	0.78	0.27	-0.13	0.67	0.19	-0.36	-0.74	0.03	0.07	0.35	-0.13	0.82	0.15
Age 6-19	3.11	2.41	3.81	-0.02	-0.80	0.76	0.96	0.14	-0.82	1.11	0.77	-0.05	-0.88	0.77	0.90	0.56	-0.25	1.36	0.17	-0.92	-1.90	0.06	0.07
Age 20-45	8.89	7.37	10.40	0.87	-0.82	2.56	0.31	-0.55	-2.63	1.53	0.60	2.14	0.34	3.93	0.02	0.72	-1.01	2.46	0.41	-1.44	-3.55	0.68	0.18
Age 46-65	1.23	0.71	1.75	-0.20	-0.78	0.38	0.50	0.37	-0.34	1.09	0.31	0.43	-0.18	1.05	0.17	-0.54	-1.13	0.06	0.08	0.30	-0.43	1.03	0.42
Age >65	1.14	0.70	1.57	-0.26	-0.74	0.23	0.30	0.31	-0.29	0.90	0.31	0.06	-0.46	0.57	0.83	0.26	-0.24	0.75	0.31	-0.29	-0.90	0.31	0.35
Maternal and neonatal	8.10	6.61	9.60	0.90	-0.77	2.57	0.29	0.47	-1.58	2.53	0.65	0.31	-1.46	2.08	0.73	0.99	-0.72	2.70	0.26	-3.33	-5.41	-1.24	0.002
NCDs	3.84	2.96	4.72	-0.48	-1.47	0.50	0.34	0.65	-0.56	1.87	0.29	0.37	-0.67	1.41	0.49	-0.53	-1.54	0.48	0.30	-1.23	-2.46	-0.001	0.05
CDs	6.49	5.38	7.60	-0.94	-2.18	0.30	0.14	-0.32	-1.84	1.21	0.68	0.97	-0.34	2.29	0.15	-0.73	-2.00	0.55	0.26	-1.04	-2.59	0.51	0.19
Injuries	2.47	1.98	2.96	-0.41	-0.96	0.14	0.15	-0.27	-0.94	0.41	0.44	0.46	-0.13	1.04	0.13	-0.22	-0.78	0.35	0.45	-0.74	-1.43	-0.06	0.03
Respiratory	0.73	0.35	1.10	-0.49	-0.91	-0.08	0.02	-0.26	-0.77	0.25	0.32	1.27	0.83	1.71	<0.001	-0.70	-1.12	-0.27	0.002	-0.11	-0.63	0.41	0.68

NCDs: non-communicable disease

CDs: communicable diseases

CI: confidence interval

For peer review only

Supplementary Table 2: Time trends and step changes in daily hospital admissions before and after start of level 5 lockdown: Poisson regression models adjusted for day of week

	Pre-lockdown daily incidence rate	95% Confidence Interval		IRR pre-lockdown (per week)	95% Confidence Interval		P value	IRR at lockdown	Confidence Interval		P value	IRR post-lockdown (per week)	95% Confidence Interval		P value	P value of interaction term
All admissions	16.06	14.30	18.03	0.990	0.977	1.003	0.14	1.06	0.95	1.19	0.31	1.004	1.000	1.007	0.04	0.05
Male >19	1.71	1.24	2.36	0.94	0.91	0.97	<0.001	1.50	1.10	2.06	0.01	1.011	1.002	1.020	0.01	<0.001
Female>19	9.23	7.92	10.76	1.01	0.99	1.02	0.57	1.05	0.91	1.21	0.51	1.004	1.000	1.009	0.06	0.92
Age <1	1.12	0.71	1.75	0.96	0.92	1.004	0.08	0.74	0.48	1.16	0.19	0.99	0.98	1.01	0.45	0.16
Age 1-5	0.69	0.40	1.19	0.94	0.89	0.998	0.04	0.88	0.51	1.52	0.65	1.00	0.98	1.02	0.85	0.05
Age 6-19	3.50	2.65	4.62	1.02	0.99	1.05	0.26	0.96	0.73	1.26	0.76	1.00	0.99	1.01	0.53	0.21
Age 20-45	9.17	7.86	10.69	1.00	0.98	1.02	0.91	1.08	0.93	1.25	0.29	1.005	1.0003	1.01	0.04	0.69
Age 46-65	1.01	0.67	1.52	0.96	0.92	1.01	0.10	1.34	0.91	1.96	0.14	1.006	0.995	1.018	0.26	0.06
Age >65	0.82	0.51	1.32	0.95	0.90	1.0003	0.05	1.23	0.77	1.96	0.39	1.012	0.998	1.026	0.08	0.02
Maternal and neonatal	9.67	8.27	11.30	1.03	1.01	1.05	0.01	1.01	0.87	1.18	0.86	0.997	0.992	1.002	0.21	0.003
NCDs	3.27	2.57	4.18	0.97	0.95	1.001	0.06	1.26	0.998	1.58	0.05	0.995	0.988	1.002	0.19	0.14
CDs	5.52	4.55	6.00	0.98	0.96	0.998	0.04	1.07	0.89	1.28	0.47	0.997	0.991	1.003	0.29	0.08
Injuries	1.83	1.26	2.00	0.95	0.91	0.99	0.02	1.29	0.88	1.89	0.19	0.986	0.974	0.999	0.04	0.09
Respiratory	0.40	0.23	0.71	0.92	0.87	0.97	0.004	1.25	0.73	2.12	0.41	1.02	1.002	1.03	0.03	0.001

NCDs: non-communicable disease

CDs: communicable diseases

For peer review only

Supplementary Table 3. Time trends and step changes in daily hospital admissions before and after start of level 5 lockdown: linear regression models adjusted for day of week

	Average admissions day before lockdown	95% Confidence Interval		Change in daily admissions/week pre-lockdown	95% Confidence Interval		P value	Change in daily admissions at lockdown	95% Confidence Interval		P value	Change in daily admissions/week post-lockdown	95% Confidence Interval		P value	P value of interaction term
All admissions	15.71	12.75	18.66	-0.20	-0.55	0.15	0.26	1.17	-1.76	4.10	0.43	0.08	-0.02	0.17	0.10	0.13
Male >19	1.42	0.42	2.43	-0.18	-0.30	-0.06	0.003	1.08	0.08	2.08	0.03	0.04	0.00	0.07	0.02	0.001
Female>19	9.03	6.82	11.24	0.06	-0.20	0.32	0.67	0.58	-1.61	2.77	0.60	0.05	-0.02	0.12	0.13	0.99
Age <1	1.11	0.60	1.63	-0.07	-0.13	-0.01	0.02	-0.31	-0.82	0.20	0.23	-0.01	-0.02	0.01	0.48	0.04
Age 1-5	0.64	0.16	1.13	-0.07	-0.12	-0.01	0.02	-0.05	-0.53	0.43	0.85	0.001	-0.01	0.02	0.87	0.03
Age 6-19	3.49	2.49	4.50	0.06	-0.06	0.18	0.31	-0.14	-1.13	0.86	0.79	-0.01	-0.04	0.02	0.57	0.26
Age 20-45	8.92	6.73	11.11	0.01	-0.25	0.27	0.95	0.92	-1.26	3.09	0.41	0.06	-0.01	0.13	0.08	0.70
Age 46-65	0.82	0.06	1.57	-0.07	-0.16	0.02	0.13	0.50	-0.24	1.24	0.19	0.01	-0.01	0.04	0.28	0.08
Age >65	0.72	0.10	1.34	-0.07	-0.14	0.00	0.07	0.24	-0.37	0.85	0.44	0.02	0.00	0.04	0.09	0.03
Maternal and neonatal	9.74	7.58	11.89	0.27	0.01	0.52	0.04	0.23	-1.91	2.36	0.83	-0.03	-0.10	0.03	0.32	0.03
Non-communicable diseases	3.07	1.79	4.34	-0.13	-0.28	0.02	0.09	1.11	-0.15	2.38	0.08	-0.02	-0.06	0.02	0.24	0.19
Communicable diseases	5.38	3.78	6.97	-0.18	-0.37	0.01	0.06	0.54	-1.04	2.12	0.50	-0.02	-0.07	0.03	0.37	0.11
Injuries	1.83	1.13	2.54	-0.11	-0.19	-0.02	0.01	0.46	-0.24	1.16	0.20	-0.02	-0.04	0.001	0.06	0.06
Respiratory	0.14	-0.42	0.71	-0.09	-0.16	-0.03	0.01	0.23	-0.32	0.79	0.41	0.02	0.000	0.036	0.04	0.001

For peer review only

Supplementary Table 4: Risk of death among admissions: logistic regression models with and without adjustment for sex, age category and diagnosis.*

	Odds ratio	95% Confidence Interval		P value
**Model 1, not adjusted for case mix**				
Weekly change in risk of death pre-lockdown	1.03	0.97	1.09	0.37
Step change in risk of death at lockdown	0.49	0.29	0.82	0.01
Weekly change in risk of death post-lockdown	1.03	1.01	1.04	0.002*
**Model 2, adjusted for case mix**				
Weekly change in risk of death pre-lockdown	1.10	1.02	1.18	0.01
Step change in risk of death at lockdown	0.31	0.17	0.56	<0.001
Weekly change in risk of death post-lockdown	1.03	1.01	1.05	0.001*
Female	0.73	0.56	0.94	0.02
Age	1.02	1.02	1.03	<0.001
Communicable disease	2.33	1.46	3.71	<0.001
Neoplasm	3.32	1.52	7.26	0.003
Blood disorder	1.96	1.01	3.83	0.05
Endocrine	0.95	0.48	1.90	0.89
Mental health	0.41	0.13	1.35	0.14
Neurological	2.33	0.99	5.49	0.05
Otological	2.59	0.33	20.63	0.37
Circulatory	3.11	1.84	5.24	<0.001
Respiratory	2.47	1.49	4.08	<0.001
Gastroenterology	2.75	1.42	5.31	0.003
Dermatology	0.73	0.30	1.80	0.49
Musculoskeletal	0.49	0.07	3.62	0.49
Renal and urology	1.48	0.78	2.82	0.23
Breast and gynaecology	0.27	0.04	2.06	0.21
Maternal	1.00	0.00	0.00	<0.001
Neonatal	2.31	1.15	4.66	0.02
Injuries	0.31	0.09	1.04	0.06

* Lockdown-time interaction: P=0.325 for model 1 and P=0.126 for model 2

For peer review only

Supplementary Figure 1. LOWESS curves of daily admissions by age and sex, 2019-2020

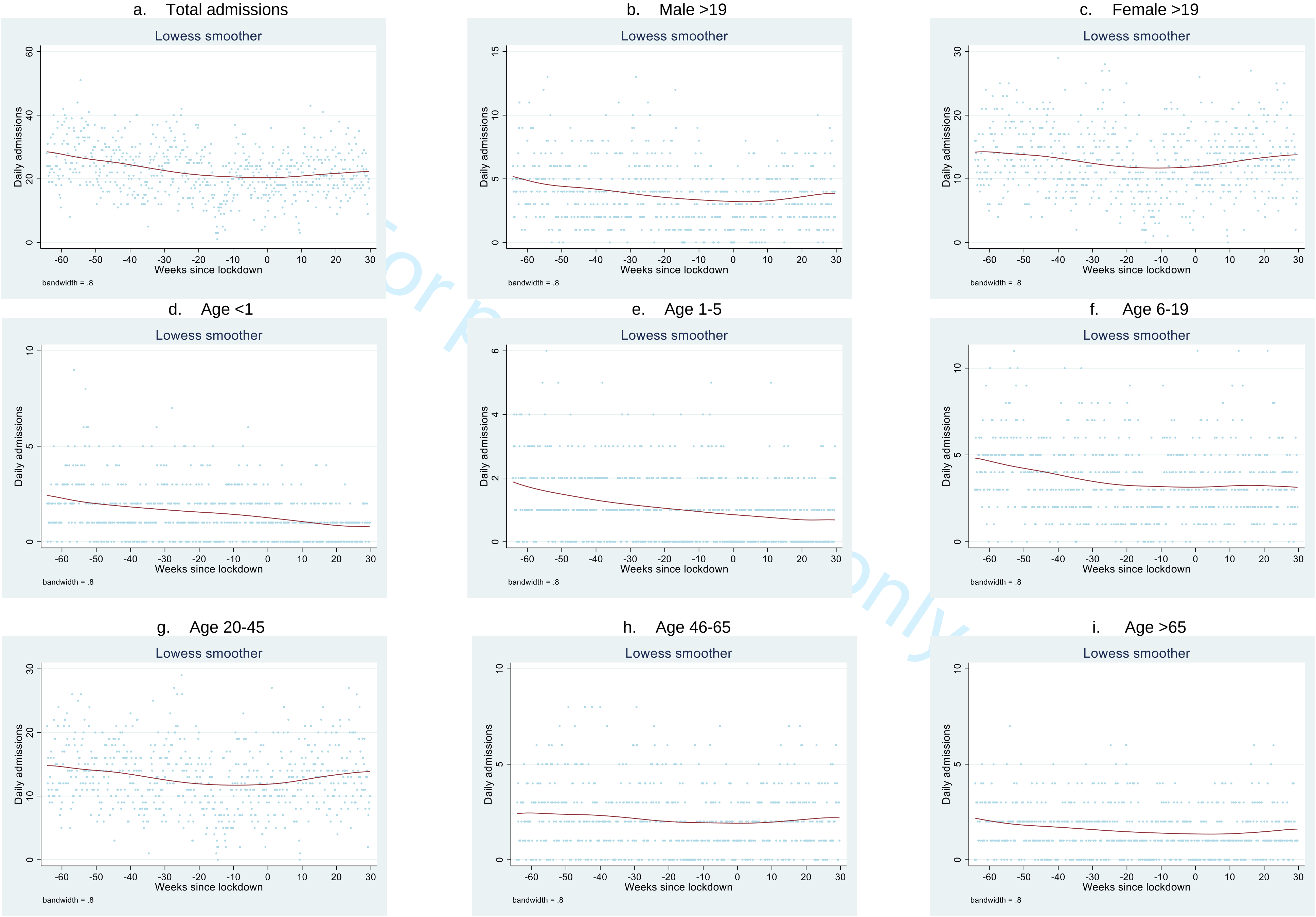

For peer review only

Supplementary Figure 2. LOWESS curves of daily admissions by diagnostic category, 2019-2020

a. Maternal and neonatal

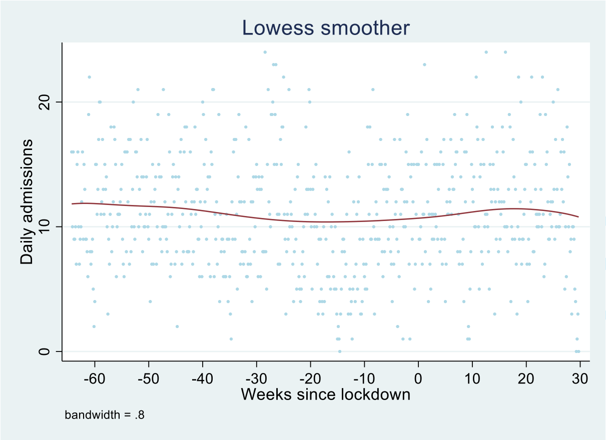

b. Non-communicable diseases

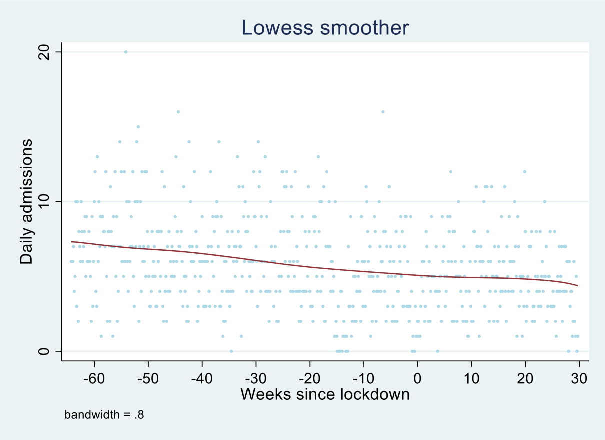

c. Communicable diseases

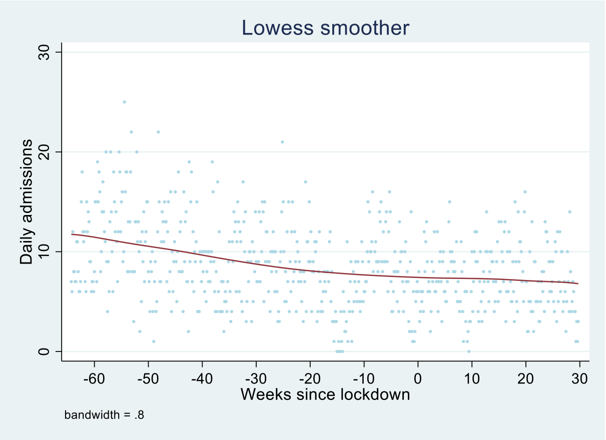

d. Injuries

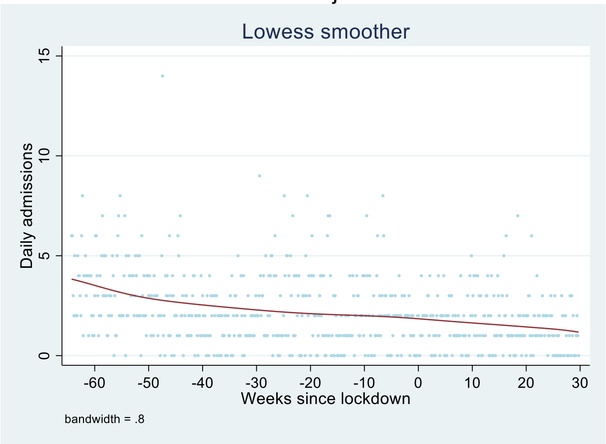

e. Respiratory

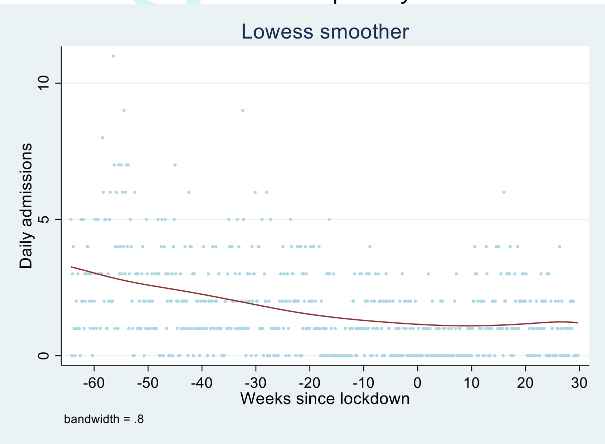

Supplementary Figure 3. LOWESS curve of probability of death by week of admission

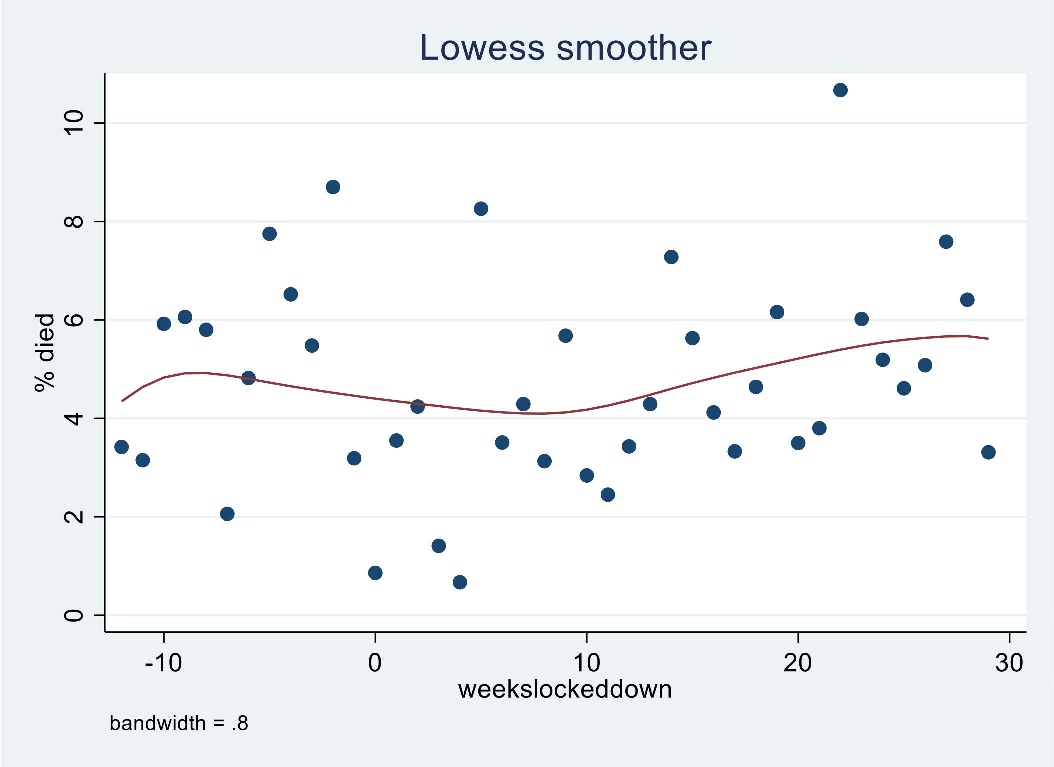

For peer review only - http://bmjopen.bmj.com/site/about/guidelines.xhtml

STROBE 2007 (v4) Statement—Checklist of items that should be included in reports of *cohort studies*

Section/Topic	Item #	Recommendation	Reported on page #
**Title and abstract**	1	(*a*) Indicate the study's design with a commonly used term in the title or the abstract	1
		(*b*) Provide in the abstract an informative and balanced summary of what was done and what was found	2
**Introduction**			
Background/rationale	2	Explain the scientific background and rationale for the investigation being reported	4
Objectives	3	State specific objectives, including any prespecified hypotheses	5
**Methods**			
Study design	4	Present key elements of study design early in the paper	5-6
Setting	5	Describe the setting, locations, and relevant dates, including periods of recruitment, exposure, follow-up, and data collection	5-6
Participants	6	(*a*) Give the eligibility criteria, and the sources and methods of selection of participants. Describe methods of follow-up	5-6
		(*b*) For matched studies, give matching criteria and number of exposed and unexposed	
Variables	7	Clearly define all outcomes, exposures, predictors, potential confounders, and effect modifiers. Give diagnostic criteria, if applicable	5-6
Data sources/ measurement	8*	For each variable of interest, give sources of data and details of methods of assessment (measurement). Describe comparability of assessment methods if there is more than one group	*5-6*
Bias	9	Describe any efforts to address potential sources of bias	5-6
Study size	10	Explain how the study size was arrived at	5
Quantitative variables	11	Explain how quantitative variables were handled in the analyses. If applicable, describe which groupings were chosen and why	5-7
Statistical methods	12	(*a*) Describe all statistical methods, including those used to control for confounding	5-7
		(*b*) Describe any methods used to examine subgroups and interactions	5-7
		(*c*) Explain how missing data were addressed	8
		(*d*) If applicable, explain how loss to follow-up was addressed	n/a
		(*e*) Describe any sensitivity analyses	6-7
**Results**			

For peer review only

Participants	13*	(a) Report numbers of individuals at each stage of study—eg numbers potentially eligible, examined for eligibility, confirmed eligible, included in the study, completing follow-up, and analysed	7
		(b) Give reasons for non-participation at each stage	n/a
		(c) Consider use of a flow diagram	n/a
Descriptive data	14*	(a) Give characteristics of study participants (eg demographic, clinical, social) and information on exposures and potential confounders	5, 7-8
		(b) Indicate number of participants with missing data for each variable of interest	n/a
		(c) Summarise follow-up time (eg, average and total amount)	n/a
Outcome data	15*	Report numbers of outcome events or summary measures over time	7
Main results	16	(*a*) Give unadjusted estimates and, if applicable, confounder-adjusted estimates and their precision (eg, 95% confidence interval). Make clear which confounders were adjusted for and why they were included	8-11, supplementary tables
		(*b*) Report category boundaries when continuous variables were categorized	11
		(*c*) If relevant, consider translating estimates of relative risk into absolute risk for a meaningful time period	Supplementary tables
Other analyses	17	Report other analyses done—eg analyses of subgroups and interactions, and sensitivity analyses	8-11
**Discussion**			
Key results	18	Summarise key results with reference to study objectives	12
**Limitations**			
Interpretation	20	Give a cautious overall interpretation of results considering objectives, limitations, multiplicity of analyses, results from similar studies, and other relevant evidence	12-14
Generalisability	21	Discuss the generalisability (external validity) of the study results	14
**Other information**			
Funding	22	Give the source of funding and the role of the funders for the present study and, if applicable, for the original study on which the present article is based	19

*Give information separately for cases and controls in case-control studies and, if applicable, for exposed and unexposed groups in cohort and cross-sectional studies.

Note: An Explanation and Elaboration article discusses each checklist item and gives methodological background and published examples of transparent reporting. The STROBE checklist is best used in conjunction with this article (freely available on the Web sites of PLoS Medicine at http://www.plosmedicine.org/, Annals of Internal Medicine at http://www.annals.org/, and Epidemiology at http://www.epidem.com/). Information on the STROBE Initiative is available at www.strobe-statement.org.

For peer review only
